# Computational investigation of Amyloid-β-induced location- and subunit-specific disturbances of NMDAR at hippocampal dendritic spine in Alzheimer’s disease

**DOI:** 10.1371/journal.pone.0182743

**Published:** 2017-08-24

**Authors:** Jingyi Liang, Don Kulasiri, Sandhya Samarasinghe

**Affiliations:** Centre for Advanced Computational Solutions (C-fACS), Lincoln University, Christchurch, New Zealand; University of South Carolina School of Medicine, UNITED STATES

## Abstract

In Alzheimer’s disease (AD), dysregulation of intracellular Ca^2+^ signalling has been observed as an early event prior to the presence of clinical symptoms and is believed to be a crucial factor contributing to AD pathogenesis. Amyloid-β oligomers (AβOs) disturb the N-methyl-D-aspartate receptor (NMDAR)-mediated postsynaptic Ca^2+^ signalling in response to presynaptic stimulation by increasing the availability of extracellular glutamate as well as directly disturbing the NMDARs. The abnormal Ca^2+^ response can further lead to impairments in long-term potentiation (LTP), an important process in memory formation. In this study, we develop a mathematical model of a CA1 pyramidal dendritic spine and conduct computational experiments. We use this model to mimic alterations by AβOs under AD conditions to investigate how they are involved in the Ca^2+^ dysregulation in the dendritic spine. The alterations in glutamate availability, as well as NMDAR availability and activity, are studied both individually and globally. The simulation results suggest that alterations in glutamate availability mostly affect the synaptic response and have limited effects on the extrasynaptic receptors. Moreover, overactivation of extrasynaptic NMDARs in AD is unlikely to be induced by presynaptic stimulation, but by upregulation of the resting level of glutamate, possibly resulting from these alterations. Furthermore, internalisation of synaptic NR2A-NMDAR shows greater damage to the postsynaptic Ca^2+^ response in comparison with the internalisation of NR2B-NMDARs; thus, the suggested neuroprotective role of the latter is very limited during synaptic transmission in AD. We integrate a CaMKII state transition model with the Ca^2+^ model to further study the effects of alterations of NMDARs in the CaMKII state transition, an important downstream event in the early phase of LTP. The model reveals that cooperation between NR2A- and NR2B-NMDAR is required for LTP induction. Under AD conditions, internalisation of membrane NMDARs is suggested to be the cause of the loss of synapse numbers by disrupting CaMKII-NMDAR formation.

## Introduction

Alzheimer’s disease (AD) is characterised by progressive and irreversible loss of memory and cognitive functions, but the exact pathophysiology and pathogenesis of the disease are still unknown [1]. Calcium (Ca^2+^) dysregulation has been observed in the brains of AD patients before the presence of overt clinical symptoms or the development of the classic biological hallmarks of amyloid plaques and neurofibrillary tangles [2, 3]. Genetic studies have also revealed altered levels of the genes and proteins related to intracellular Ca^2+ ^signalling pathways in AD cells [4, 5]. The Ca^2+ ^hypothesis of AD, which was first proposed by Khachaturian, and many subsequent experimental studies have suggested that the sustained disturbances in intracellular Ca^2+ ^signalling contribute to the major symptoms of AD and may be the predominant cause of the neurodegeneration in AD [6–8].

Amyloid-β oligomers (AβOs) have been reported to disturb neuronal Ca^2+^ by targeting various components of the Ca^2+^ signalling network, ranging from glutamatergic neurotransmission, membrane channels and pumps, to intracellular Ca^2+^ sources [9–11]. The overall effect leads to abnormal intracellular Ca^2+^ transients, elevation in the basal level of cytosolic Ca^2+^ and, ultimately, intracellular Ca^2+^ [12]. Alteration of intracellular Ca^2+^ signalling is a key upstream event in AD pathophysiology that initiates and accelerates other severe downstream events, such as amyloid plaque deposition and neuronal apoptosis [9, 13].

**Fig 1:**
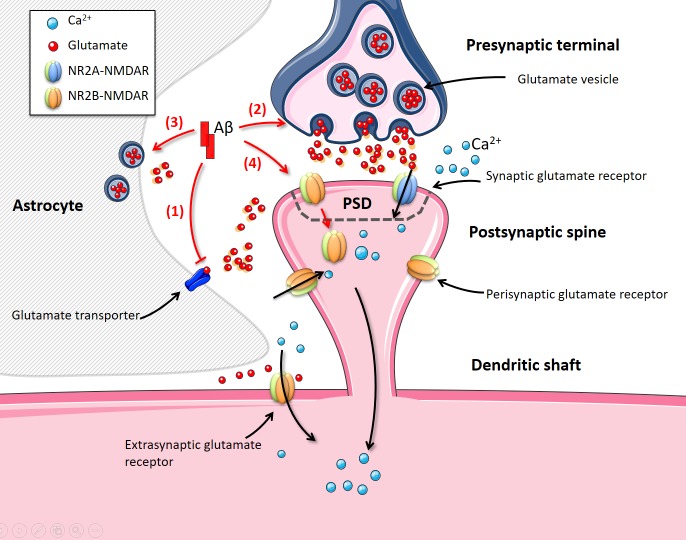
Disturbances in glutamatergic synaptic transmission by AβO in AD. Referring to the arrow labels: (1) AβO inhibits glutamate clearance by the glutamate transporters; and (2) and (3) AβO promotes glutamate vesicle release from the presynaptic terminal and ambient astrocytes, respectively; and (4) AβO also mediates in the internalisation of the surface receptors at the synaptic site. (1), (2) and (3) result in an increase in the extracellular glutamate concentration and, ultimately, may lead to the over activation of synaptic glutamate receptors or of receptors at distant locations from the release site. In contrast, (4) leads to a decrease in functional synaptic receptors that may depress synaptic activity. This figure is produced using Servier Medical Art (http://www.servier.com/Powerpointimage-bank).

There are two types of glutamate receptors in the pyramidal neurons of the hippocampus: the N-methyl-D-aspartate receptor (NMDAR) and the α-amino-3-hydroxy-5-methyl-4-isoxazolepropionic acid receptor (AMPAR) at the excitatory synapses [14]. NMDARs are located at both the synaptic active zone and the extrasynaptic region [15] (Fig 1), which includes a perisynaptic zone, a membrane area that surrounds the synaptic zone, and an extrasynaptic zone containing the dendritic spine neck, the dendritic shaft, and the neuron body. NMDARs play roles in Ca^2+^ signalling as plasma membrane Ca^2+^ channels and in the formation of the Ca^2+^ /calmodulin-dependent protein kinase II (CaMKII)-NMDAR complex, a critical modulator in long-term potentiation (LTP) induction in the postsynaptic density (PSD) [16, 17]. NMDAR is a heterotetramer mostly composed of two NR1 subunits and two NR2 subunits [18, 19]. In the hippocampus, NR2 subunits express dominantly as NR2A and NR2B [20]. The NR2 subunit composition of NMDARs determines their key properties, such as glutamate affinity, open probability and desensitisation rate [20–22] (see S1 Appendix for definitions and explanations), which makes the ratio of NR2A to NR2B an important factor in the Ca^2+^ response and synaptic plasticity [23]. In mature synapses, NR2A-containing NMDARs (NR2A-NMDARs) are predominant at the synaptic sites, and comprise of approximately 60% of the total synaptic NMDARs [24]. In contrast, NMDARs located outside the synaptic region are mainly NR2B-containing NMDARs (NR2B-NMDARs) [24]. Synaptic and extrasynaptic NMDARs are proposed to play opposite physiological roles in mediating intracellular signalling and death pathways: the activation of synaptic NMDARs is shown to promote cell survival, whereas stimulation of the extrasynaptic NMDARs contributes to cell death (see [25] for a review).

AβO can affect glutamatergic synaptic transmission by increasing the availability of extracellular glutamate as well as directly disturbing the NMDARs (Fig 1) (see Section 2 of S1 Appendix). Experimental observations, as discussed briefly in Section 2 of S1 Appendix, show the paradoxical effects of AβOs on the Ca^2+^ dynamics of the postsynaptic neurons which lead to different interpretations of disturbances in the downstream events that are mediated by the cytosolic Ca^2+^ levels. Upregulation in the availability of glutamate to NMDARs may be the major reason for the excitotoxicity and Ca^2+^ overload observed in AD, suggesting that AβO is a factor in inducing the enhanced excitotoxicity [11, 26–29]. The loss of synaptic NMDARs may inhibit NMDAR-dependent LTP [30, 31], contributing to the depression of glutamatergic transmission and reductions in memory formation. However, to some extent, the loss of synaptic NMDARs may also be a neuroprotective mechanism against the glutamate-induced neurotoxicity and excessive influx of Ca^2+^ [32, 33]. The effects of these alterations are studied individually in transgenic animal models or by injecting a high concentration of Aβ into healthy animals or cells [32, 34–40]. However, the use of different experiment materials or experimental protocols from different research groups can lead to controversial results for AβO disturbances (see reviews in [41–43]). A mathematical model of Ca^2+^ dynamics in the dendritic spine with presynaptic stimulations as inputs, will provide useful insights into the effects of the above disturbances at different levels in AD, both individually and collectively.

In this paper, we present an integrated mathematical model of an average CA1 pyramidal dendritic spine that includes location- and NR2 subunit-specific characteristics of NMDARs to understand the AβO–induced dysregulation of intracellular Ca^2+^ and its relationship to the balance between synaptic and extrasynaptic NMDARs in the dendritic spine of pyramidal neurons. We use the model to mimic several types of disturbances of AβO that have been proposed to be critical to the pathogenesis of AD (see Section 2 of S1 Appendix). The model shows that AβO-dependent disturbances on synaptic glutamatergic transmission mainly affect Ca^2+^ signalling in the dendritic spine and have only minor effects on Ca^2+^ signalling in the whole cell. In contrast, AβO-induced non-synaptic glutamate release and elevation in extrasynaptic glutamate concentration mainly affect the Ca^2+^ dynamics of the whole cell. Furthermore, the simulation results suggest that among all disturbances tested, the presynaptic release of glutamate is the most sensitive disturbance for NMDAR activity and Ca^2+^ response in the postsynaptic neurons.

We also extend our research on synaptic Ca^2+^ signalling to the downstream CaMKII state transition to investigate the disturbances from alterations in synaptic NMDAR in the emergence of LTP in AD: we integrate the Ca^2+^ model with a state transition model of CaMKII to study the subunit-specific roles of NMDAR in the postsynaptic response and the formation of NMDAR-mediated LTP. The model reveals that cooperation between NR2A- and NR2B-NMDAR is necessary for NMDAR-LTP. Simulation results suggest that the internalisation of synaptic NR2B-NMDAR observed in the experiments [31, 39] does not severely affect the synaptic Ca^2+^ responses, thus, it is not enough to protect against the glutamate-induced neurotoxicity in AD. Instead, loss of synaptic membrane NR2B-NMDAR will inhibit LTP induction by disrupting CaMKII-NMDAR formation.

## Methods

### Model overview

**Fig 2:**
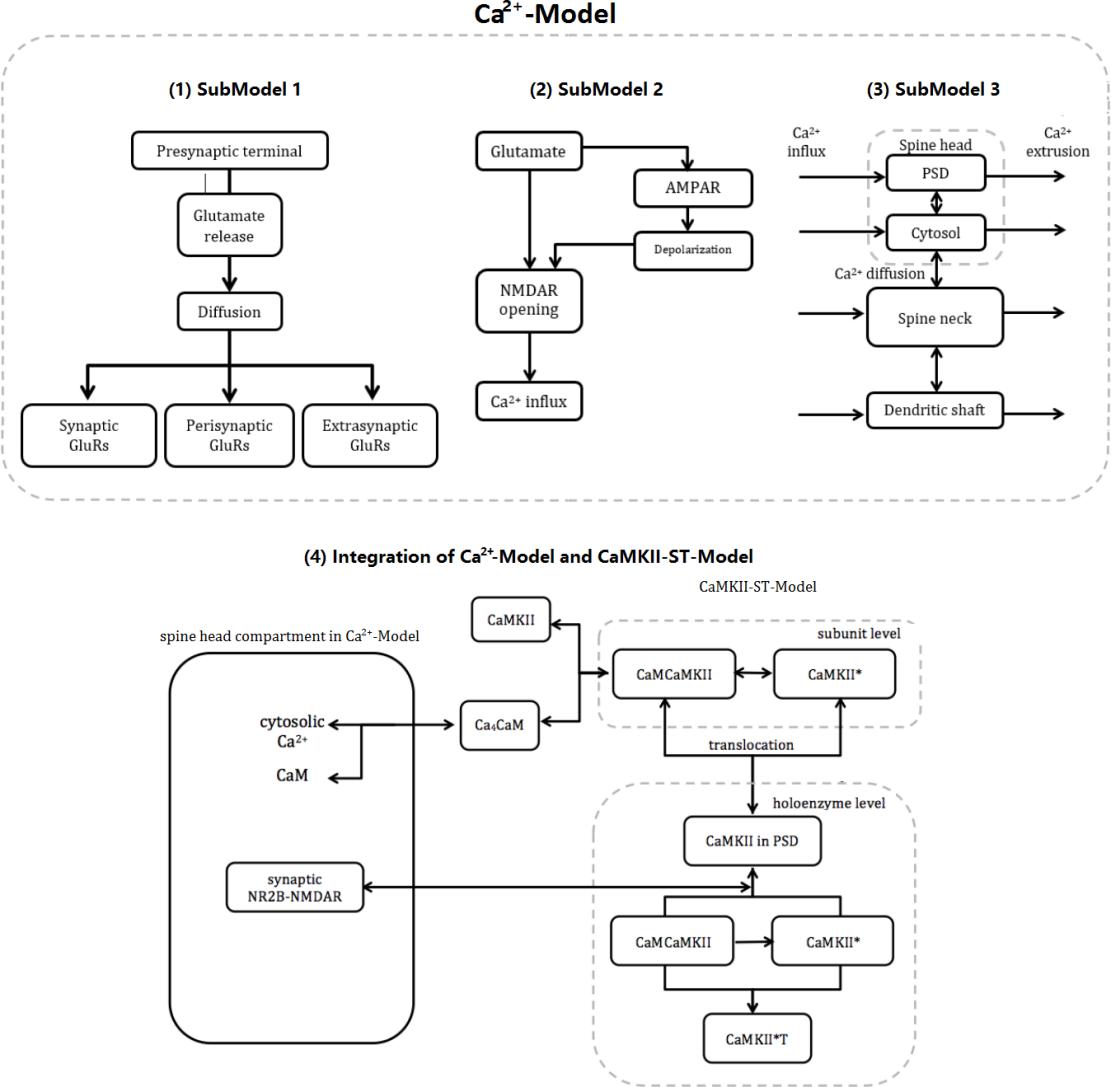
Model conceptual framework. Ca^2+^-Model consists of three submodels: (A) SubModel 1: After presynaptic stimulation, glutamate is released from the presynaptic terminal into the synaptic cleft. It is then diffuses across the synaptic cleft and into the extrasynaptic space. Through diffusion, glutamate can bind to the glutamate receptors at different locations; (B) SubModel 2: Glutamate receptor activation. NMDAR and AMPAR are the most common ionotropic glutamate receptors. NMDAR is the major Ca^2+^ channel; Ca^2+^ influx through NMDAR requires both the binding of glutamate to NMDAR and the removal of its Mg^2+^ blockage. The latter can be achieved by membrane depolarisation after the activation of AMPARs; (C) SubModel 3: A four-compartment model of the dendritic spine and its adjacent dendritic shaft that includes the mechanisms for Ca^2+^ influx, extrusion and buffering in each compartment and diffusion between the two neighbouring compartments; and (D) Schematics of the model integration of Ca^2+^-Model with CaMKII-ST-Model [44].

We construct a mathematical model of Ca^2+^ dynamics (denoted as Ca^2+^-Model) in the dendritic spine in response to presynaptic stimulation, which is carefully developed according to the characteristics of the CA1 pyramidal dendritic spine. Ca^2+^-Model integrates three aspects: (1) SubModel 1: action potential (AP)-induced presynaptic glutamate release and diffusion; (2) SubModel 2: glutamate receptor activation; and (3) SubModel 3: Ca^2+^ dynamics within a single dendritic spine and its parent dendrite shaft (Fig 2A–2C). To further study the effects of NMDAR alterations in their roles in downstream events, we extend Ca^2+^-Model with a CaMKII state transition model (denoted as CaMKII-ST-Model) by He et al. [44] (Fig 2D). All simulations are performed using Matlab software, with the built-in solver ode15.

### Ca^2+^-Model development

#### SubModel 1: A model of glutamate release, uptake and diffusion

We simulate the glutamate release from the presynaptic terminal, and its diffusion inside the synaptic cleft and in the extrasynaptic space, based on the model by Rusakov and Kullmann [45]. A schematic of SubModel 1 in two-dimensions is given in Figure A in S2 Appendix. The dendritic spine head and the presynaptic terminal are configured as two opposite hemispheres with the same radius and there is no glutamate diffusion within them. The synaptic cleft is a flat cylinder between these two hemispheres, with a height of 20 nm. We take 0.1 μm^3^ as the volume of the spine head to represent the average size of spine [46], which gives a radius of 363 nm for the synaptic cleft and the two opposite hemispheres. The extrasynaptic space is a spherically isotropic porous medium surrounded by the two hemispheric obstacles.

The concentration of glutamate in a glutamate vesicle is about 100 mM [47], which corresponds to about 1500 glutamate molecules. Therefore, we assume that a presynaptic stimulation pulse can induce the release of 1500 glutamate molecules from a point site in the centre of the presynaptic terminal surface (see Section 1 of S2 Appendix). Once released, glutamate molecules diffuse through the flat cylindrical cleft with an effective glutamate diffusion coefficient (D_Glu_), and then escape from the cleft into the extrasynaptic space, where D_Glu_ is reduced by a tortuosity factor λ (${D_{Glu}}^{*}=\frac{D_{Glu}}{\lambda^{2}}$). The uptake of glutamate is governed by glutamate transporters in astrocytes in the extrasynaptic space. In the extrasynaptic space, glutamate transporters are distributed homogeneously at a concentration (B_total_) of 0.5 mM [48] (see Section 1 of S2 Appendix). To track the glutamate after release at different locations, we define PSD or synaptic, perisynaptic and extrasynaptic sites as follows: the surface of PSD region and perisynaptic zone are set from 0 to 150 nm and 365 nm away from the centre of the postsynaptic surface, respectively [49] (Figure A (1) and (2) in S2 Appendix). The extrasynaptic space is set beyond the outside border of the perisynaptic zone and the extrasynaptic receptors are located at the dendritic shaft.

#### SubModel 2: A model of NMDAR and AMPAR activation

Glutamate receptors are located at the synaptic, perisynaptic and extrasynaptic membrane surfaces with varying numbers (Table B in S2 Appendix). Each receptor is independent of each other and the local glutamate concentration received by each receptor depends on its distance from the release site and is calculated by SubModel 1. We simulate the state transition of a single NMDAR and AMPAR using an NR2 subtype specific, eight-state kinetic model [50] and a seven-state model [51], respectively (Figure B in S2 Appendix). The reaction rate constants are given in Table B in S2 Appendix.

Based on the experimental observations [24, 25, 49, 52, 53], the distributions of NMDARs are: 12 synaptic NR2A-NMDARs, 8 synaptic NR2B-NMDAR, 3 perisynaptic NR2B-NMDAR, and 8 extrasynaptic NR2B-NMDAR. The number of AMPARs depends on the spine geometry and is positively correlated with PSD size [49, 54]. We assume that the functional AMPARs are homogeneously located in the membrane of PSD and at the dendritic shaft with different densities, and there is no AMPAR in the rest of the spine membrane [55]. The number of AMPARs per spine is critical for the generation of the temporary depolarisation of the postsynaptic membrane potential after stimulation; this is also called the excitatory postsynaptic potential (EPSP). Based on the experiments [56, 57], the EPSP amplitude at the synaptic site is lower than 5 mV after a single synaptic stimulation. We estimate the number of synaptic AMPARs based on the established experimental data [58, 59], to generate the expected EPSP amplitude (see Section 5 of S2 Appendix). The neck conductance we use in the simulation is 157 MΩ, representing the resistance of a medium sized spine neck of CA1 pyramidal neurons [59]. The number of extrasynaptic AMPARs (eAMPARs) at the dendritic shaft is low and stable, and is calculated based on the membrane surface area (see Section 5 of S2 Appendix) [55]. The removal of the Mg^2+^ blockage of NMDARs and the activation of voltage-dependent calcium channel (VDCC) depend on the membrane depolarisation after stimulation. We build an electrical model of a single spine and its adjacent dendritic shaft to capture the dynamics of the membrane potential (see Section 3 of S2 Appendix for the model details).

#### SubModel 3: A compartmental model of a dendritic spine and shaft

We construct a four-compartment model to represent a pyramidal neuron dendritic spine and its adjacent dendritic shaft. The four compartments are PSD, cytosol, spine neck and dendritic shaft (Figure D in S2 Appendix). The geometry of the spine is consistent with that of SubModel 1 and SubModel 2 (Table A in S2 Appendix). The spine head is assumed to be a hemisphere and is divided into PSD and cytosol compartments. The PSD compartment is a cylinder attached to the postsynaptic membrane and the rest of the spine head is in the cytosol compartment. PSD occupies 10% of the total volume of the spine head [60, 61]. The spine neck is represented as a long thin cylinder, which is coaxial with the spine head. The dendritic shaft is another cylinder that is attached to the bottom of the spine neck with a radius of 0.5 μm and a length of 1 μm (details of this compartmental model are in Section 4 of S2 Appendix).

### Parameter calibration and estimation

The simulation temperature used in this paper is 34°C, a near-physiological temperature widely used in experiments and computational simulations [46, 62]. All temperature-dependent rate constants are adjusted according to their temperature coefficients (Q10), as listed in Table 1.

**Table 1:** Q10 values for biological processes. Q10 measures the degree of temperature dependence when increasing the temperature by 10°C [63].

Biological processes	Q10	Biological processes	Q10
Diffusion	1.3	Glutamate transporter kinetics	3
NMDAR kinetics	3	NMDAR conductance	1.6
AMPAR kinetics	2.4	AMPAR conductance	1.5
VDCC gating kinetics	3	VDCC conductance	1.5
Pump kinetics	3	Buffer kinetics	2.15

The values of the ten uncertain parameters of Ca^2+^-Model are estimated using Markov Chain Monte Carlo (MCMC) [64, 65]: the density for VDCC, the densities for two membrane Ca^2+^ pumps, PMCA and NCX, the concentration of the endogenous immobile buffer and its binding and unbinding rates in the dendritic and spine locations, respectively. The parameter estimation is based on the experimental observations by Sabatini, Oertner et al. [46]. They estimated the Ca^2+^dynamics at the dendritic spines of the CA1 pyramidal neurons and their parent dendrites by a single backpropagation of action potentials (bAP) in the absence of exogenous buffers (Ca^2+^ indicator) and the washout of mobile buffers. The results correspond to spines and small dendrites with surface-to-volume ratios of 4–20 μm^-1^ and 1–4 μm^-1^, respectively [46]. The geometry of our model lies well within these ranges. The details of MCMC estimation are discussed in Section 6 of S2 Appendix.

#### Model performance

The control condition is defined as the condition without AβO-disturbances and is simulated using standard values of parameters listed in Section 1–6 of S2 Appendix. We investigate the model performance in response to presynaptic stimulation (see Section 7 of S2 Appendix for a detailed explanation) based on glutamate profile, the open fraction of receptors at different locations and, consequently, the Ca^2+^ dynamics in the spine head and its parent dendritic shaft. The model performance under control conditions in response to various stimulation protocols is given in Section 8 of S2 Appendix.

### Integration of Ca^2+^-Model and CaMKII-ST-Model

CaMKII-ST-Model developed by He et al. [44] simulates the formation of CaMKII-NMDAR complex in PSD in response to presynaptic stimulation. It consists of a series of key events induced by the Ca^2+^ influx through NMDAR after stimulation. Ca_4_CaM complex and postsynaptic NR2B-NMDAR are two linking factors between Ca^2+^-Model and CaMKII-ST-Model (Fig 2D). The details of the model integration are in Section 9 of S2 Appendix.

## Results

We mimic the AβO-dependent disturbances on glutamatergic transmission on the following aspects: the availability of glutamate to glutamate receptors and the distribution of synaptic NMDARs. Based on the experimental protocols [44, 66], we apply three types of presynaptic stimulation patterns as inputs to Ca^2+^ Model: (1) a single stimulus (1 pulse); (2) a low frequency stimulation (LFS) at 10 Hz; and a (3) high frequency stimulation (HFS) at 100 Hz (see Section 7 of S2 Appendix for the details of the stimulation protocols). To investigate the effects of these alterations on the postsynaptic response, we compare the activities of NMDAR and the dynamics of Ca^2+^ at different locations with those under the control condition.

### Simulation of AβO-dependent disturbances on glutamate transmission

#### Effects of increases in presynaptic release

We first investigate the effects of the AβO-induced release of glutamate vesicles from the presynaptic terminal [67–71]. We assume every vesicle contains the same number of glutamate molecules, therefore, the number of vesicles per release is represented by the total number of glutamate molecules. We vary the number of glutamate molecules per release from 500 to 10000 and keep other parameters at the standard values.

The simulation results show that glutamate concentrations ([Glu]_peak_) at three locations all increase linearly with the number of glutamate molecules released (S1 Fig). To examine the contribution of an increase in the presynaptic release of glutamate to the NMDAR transition, we calculate the open time of each receptor and the number of Ca^2+^ ions entering through each receptor after stimulation. We define the additional time for a receptor staying in a state (t_add_) as the difference between the total time in this state under the current condition and the time under control condition. Similarly, the additional number of Ca^2+^ ions entering (Ca^2+^_add_) through a receptor is the difference between the total number of the Ca^2+^ ion flux under the current condition and the one under control condition.

**Fig 3:**
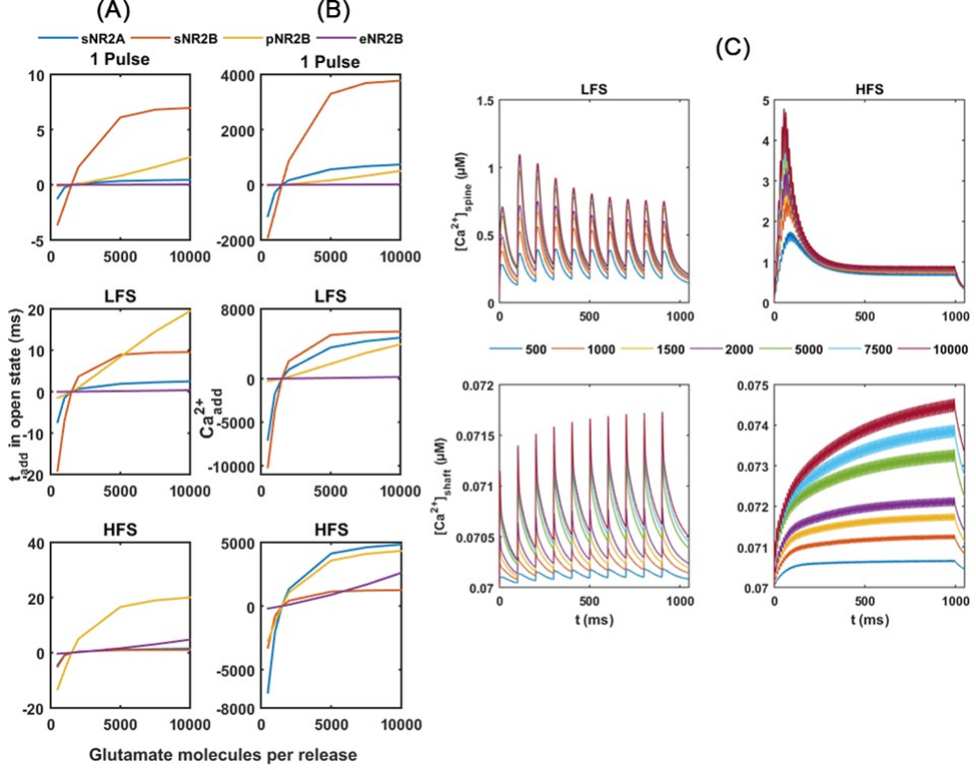
Postsynaptic responses with various numbers of glutamate molecules released. Simulation results are shown in comparison with the amount under control conditions (1500 molecules per release). (A) t_add_ per receptor; (B) Ca^2+^_add_ through NMDARs; and (C) Ca^2+^ responses in the spine head and dendritic shaft. Coloured lines represent different glutamate molecules per release. sNR2A: synaptic NR2A-NMDAR; sNR2B: synaptic NR2B-NMDAR; pNR2B: perisynaptic NR2B-NMDAR; eNR2B: extrasynaptic NR2B-NMDAR.

The simulation results show that multiple-vesicle releases have a negligible effect on the total open time of synaptic NR2A-NMDARs (Fig 3A). Even though, Ca^2+^_add_ through all synaptic NR2A-NMDARs still has made a large contribution to the total Ca^2+^_add_ into the spine head (Fig 3B). This is because the number of synaptic NR2A-NMDARs is more than that of the other receptors.

For synaptic and perisynaptic NR2B-NMDARs, multiple vesicle releases lead to increases in t_add_ in the open state under a 1 pulse stimulation and LFS. Under HFS, in contrast, only the perisynaptic NR2B-NMDARs show an increase in open time (Fig 3A). Consequently, total Ca^2+^_add_ through all perisynaptic NR2B-NMDARs increases under LFS and HFS, whereas total Ca^2+^_add_ through all synaptic NR2B-NMDARs only increases under LFS (Fig 3B).

Increases in open time and Ca^2+^ influx of synaptic and perisynaptic receptors lead to a higher peak in Ca^2+^ concentration ([Ca^2+^]_peak_) in the spine head (Fig 3C). Specifically under HFS, Ca^2+^ level rises to a peak of about 4.8 μM in 55 ms (almost a three-fold increase in Ca^2+^ level over the control condition) upon increasing the glutamate molecules per release to 10,000 (Fig 3C). After reaching the peak, Ca^2+^ levels then rapidly decrease and stay on a plateau after around 40 pulses until the end of the stimulation. The decrease in Ca^2+^ level is because of the desensitisation of NMDARs by repetitive stimulation. The plateau in Ca^2+^ level is in a range of 0.5 to 1.2 μM, and increases with the number of glutamate molecules per release.

The extrasynaptic NR2B-NMDARs are not (1 pulse stimulation and LFS) or slightly (HFS) affected by the number of glutamate molecules released in response to presynaptic stimulation (Fig 3A and 3B). In Fig 3C, the elevations in [Ca^2+^]_peak_ in the dendritic shaft by high releases of glutamate are largely the result of Ca^2+^ diffusion from the spine head, rather than Ca^2+^ influx through extrasynaptic NMDARs.

#### Effects of inhibitions of the glutamate transporter

**Fig 4:**
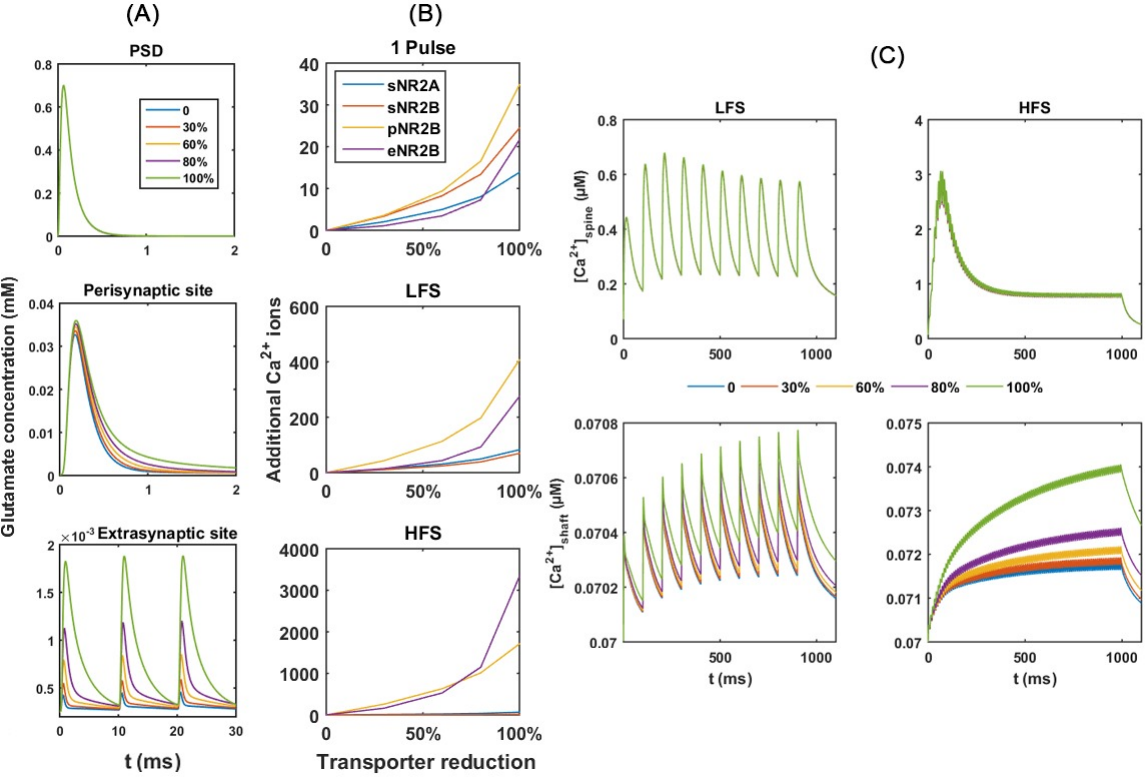
Postsynaptic responses with a reduction in transporter levels. (A) Time course of glutamate concentration at different locations. (B) Ca^2+^_add_ through NMDARs and (C) Ca^2+^ dynamics in the spine head and dendritic shaft with the reduction in B_total_ in response to three types of stimulation patterns. Percentages denote the degree of reduction in total glutamate transporter concentration (B_total_) from the standard value, 0.5 mM (0: no reduction; 1: fully reduction and B_total_ = 0). The glutamate number per release is 1500. sNR2A: synaptic NR2A-NMDAR; sNR2B: synaptic NR2B-NMDAR; pNR2B: perisynaptic NR2B-NMDAR; eNR2B: extrasynaptic NR2B-NMDAR.

Experimental evidence suggests that AβO may disturb glutamate clearance mechanisms by reducing the number of glutamate transporters [34–38].To examine if the down-regulation of glutamate transporters contributes to the abnormal opening of NMDARs, we simulate Ca^2+^-Model a total concentration of glutamate transporters (B_total_) that ranges from 0.5 mM (0% reduction) to 0 mM (100% reduction). Fig 4 shows that there is no effect on [Glu]_peak_ at the postsynaptic site from reducing B_total_. In contrast, at the perisynaptic and extrasynaptic sites, the reduction of B_total_ to 0 mM (100% reduction) increases [Glu]_peaks_ by about 3 μM and 1.5 μM, respectively, while causing a slower decay to baseline (Fig 4A). These increases lead to higher Ca^2+^_adds_ through perisynaptic NR2B-NMDARs and extrasynaptic NR2B-NMDARs, especially under HFS (Fig 4B). Consequently, inhibition in the glutamate uptake results in increases in [Ca^2+^]_peak_ in the dendritic shaft but not in the spine head (Fig 4C).

We then increase the glutamate numbers per release from 1500 to 5000 to investigate if there are any significant changes in the multi-vesicle releases. The results (S2 Fig) are consistent with the single-vesicle release in the experiment above. Therefore, the down-regulation in glutamate uptake does not directly affect the postsynaptic spine in response to the presynaptic stimulations. In contrast, this down-regulation promotes a Ca^2+^ influx through the extrasynaptic receptors in the dendritic shaft, especially under HFS.

#### Effects of increases in the resting glutamate levels in the extrasynaptic space

**Fig 5:**
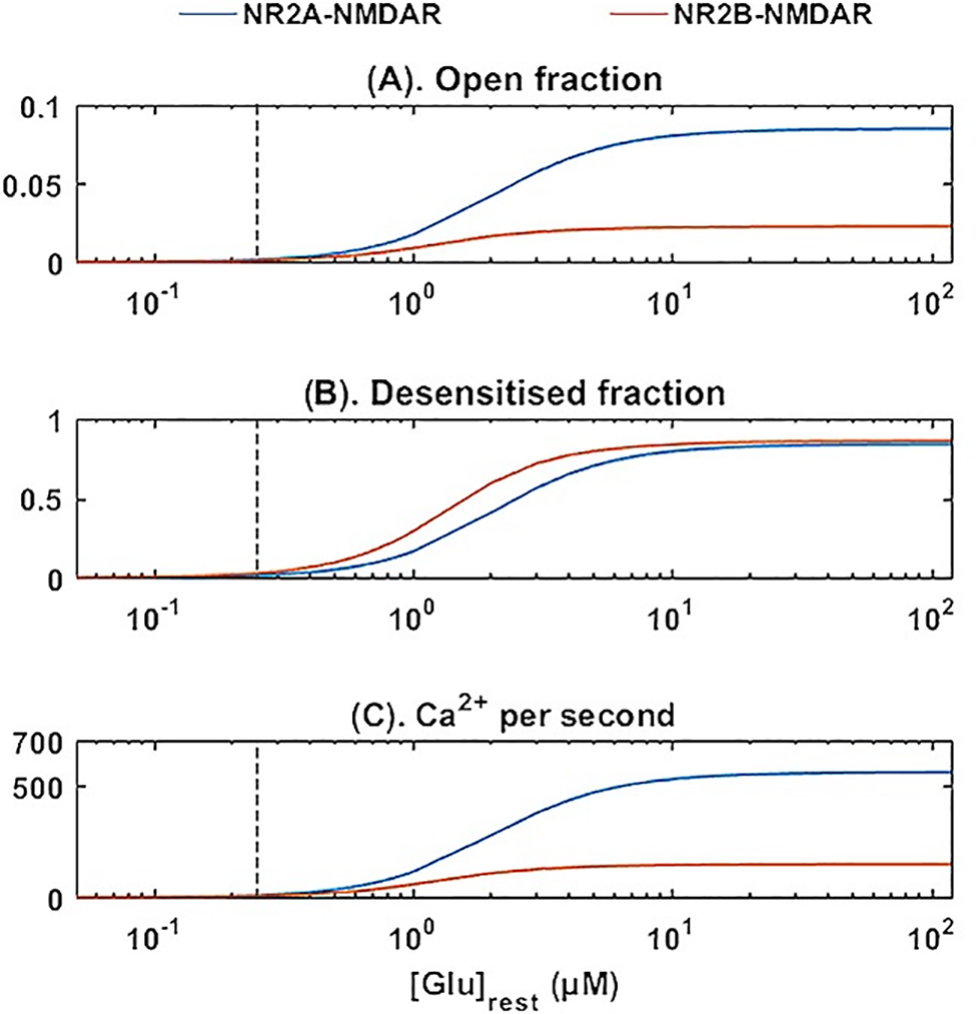
Effects of [Glu]_rest_ on receptor resting states. SubModel 2 is simulated with an increase in [Glu]_rest_, from 0.01 μM to100 μM. Coloured lines denote (A) pen fraction, (B) desensitised fraction and (C) Ca^2+^ influx for NR2A- and NR2B-NMDAR, respectively. Vertical dash lines indicate [Glu]_rest_ = 2.5 μM (control condition).

Elevations in the resting level of extrasynaptic glutamate ([Glu]_rest_) have been observed in the hippocampus of AD transgenic mice that overexpress the human amyloid precursor protein [72]. In contrast to a [Glu]_rest_ of 0.25 μM in the control mice, Talantova et al. [72] reported 0.8 and 3.3 μM in 12-month-old and 22 to 24-month-old mice, respectively. To examine how [Glu]_rest_ affects the receptors at the resting state, we simulate SubModel 2 with [Glu]_rests_ from 0.01 μM to 100 μM. The results show both NR2A- and NR2B-NMDAR reach the maximum open fractions (0.08 and 0.02, respectively) when glutamate increases to about 10 μM (Fig 5A). The fraction of NR2A- NMDAR and NR2B-NMDAR in the desensitised state approaches a maximum of 0.81 at the same time (Fig 5B). The background opening of NR2B-NMDAR by [Glu]_rest_ causes a persistent inward current and Ca^2+^ influx. When increasing [Glu]_rest_ to 10 μM, the background Ca^2+^ influx reaches a maximum of 580 (NR2A-NMDAR) and 160 (NR2B-NMDAR) Ca^2+^ ions per second, which is 5–10 Ca^2+^ ions per second lower control conditions (Fig 5C).

**Fig 6:**
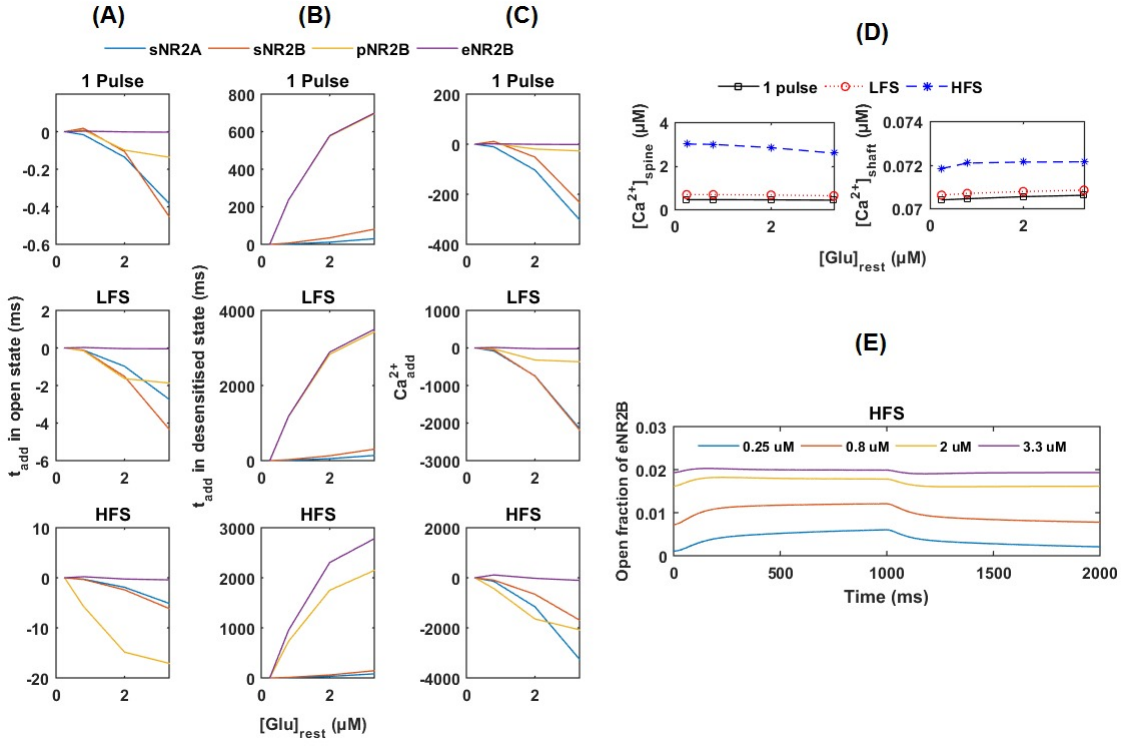
Postsynaptic responses with increasing [Glu]_rest_. (A) t_add_ in the open and (B) desensitisation states per receptor and (C) Ca^2+^_add_ through NMDARs in response to presynaptic stimulation (a single pulse stimulation, LFS and HFS) under the conditions of the increased [Glu]_rest_ compared with those under control condition. (D) Amplitudes of Ca^2+^ in the spine head and dendritic shaft at various [Glu]_rest_. (E) The fraction of extrasynaptic NR2B-NMDAR in the open state during HFS. The resting extrasynaptic glutamate concentrations are 0.25 μM in the control condition (blue line) and 0.8 μM, 2 μM and 3.3 μM, representing different stages of the disease. sNR2A: synaptic NR2A-NMDAR; sNR2B: synaptic NR2B-NMDAR; pNR2B: perisynaptic NR2B-NMDAR; eNR2B: extrasynaptic NR2B-NMDAR.

When applying different presynaptic stimulations to Ca^2+^-Model, elevation in [Glu]_rest_ slightly reduces the open time of the synaptic and perisynaptic NMDARs but not extrasynaptic NMDARs (Fig 6A). Consequently, the elevation in [Glu]_rest_ leads to fewer Ca^2+^ ions entering (Fig 6C) and decreases [Ca^2+^]_peak_ in the spine head (Fig 6D).

In contrast, the elevation in [Glu]_rest_ leads to a large increase in the desensitisation time of the perisynaptic and extrasynaptic NR2B-NMDARs (Fig 6B). The high level of [Glu]_rest_ results in a high fraction of background opening of the extrasynaptic NR2B-NMDAR and small increases in response to presynaptic stimulation (Fig 6E).

#### Effects of non-synaptic release of glutamate

AβO has been found to induce glutamate release from astrocytes in AD transgenic mice [72–75]. The astrocyte stays close to the dendritic shaft, which can potentially activate extrasynaptic glutamate receptors. We simulate the astrocytic release of glutamate by applying a brief pulse (1 to 20 ms) of 1 mM glutamate to the extrasynaptic sites. The glutamate concentration in the synaptic cleft and presynaptic site will not be affected[47].

**Fig 7:**
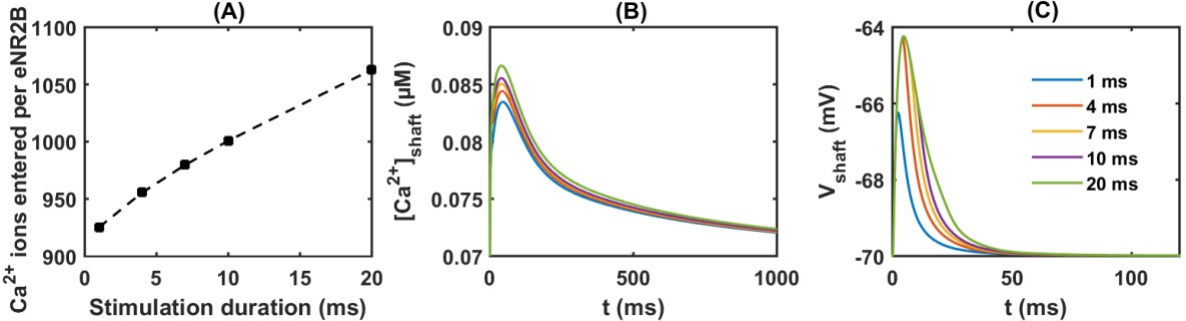
Effects of extrasynaptic NR2B-NMDAR activation by the astrocytic release of glutamate. Simulations are carried out in response a pulse of 1 mM glutamate at various time durations (1 ms, 4 ms, 7 ms, 10 ms and 20 ms). (A) Ca^2+^ ions enter through extrasynaptic NR2B-NMDARs in to the dendritic shaft. The corresponding Ca^2+^ transient and membrane depolarisations are shown in (B) and (C), respectively.

Fig 7A shows that the stimulation causes large numbers of Ca^2+^ to enter the dendritic shaft, which increases with the length of the stimulation pulse. [Ca^2+^]_peak_ in the dendritic shaft ranges from 0.083 to 0.087 μM (Fig 7B). The dendritic shaft has larger volume and fewer receptors than the spine head. Ca^2+^ ions entering through the extrasynaptic NR2B-NMDAR are largely diluted in the dendritic shaft; therefore, [Ca^2+^]_peak_ is much less than that in the spine head in response to presynaptic stimulation. Moreover, extrasynaptic AMPARs activated by the astrocytic glutamate release create a 4–6 mV depolarisation (Fig 7C). Even when increasing the stimulation time to 20 ms, it still fails to create a larger depolarisation to activate other voltage-dependent Ca^2+^ channels in the dendritic shaft membrane. This is understandable because of the low AMPAR density in the extrasynaptic site (20 receptor/μm^2^; in synaptic location up to 1000 receptor/μm^2^) [55].

### Disturbances in NMDARs in AD

#### Effects of reductions in the surface expression of synaptic NMDAR

We next investigate how inhibitions of AβO on the membrane surface expression of different receptors affect the postsynaptic response. We mimic the reduction in surface expression by decreasing the receptor number from the standard value (Table B in S2 Appendix) to zero.

**Fig 8:**
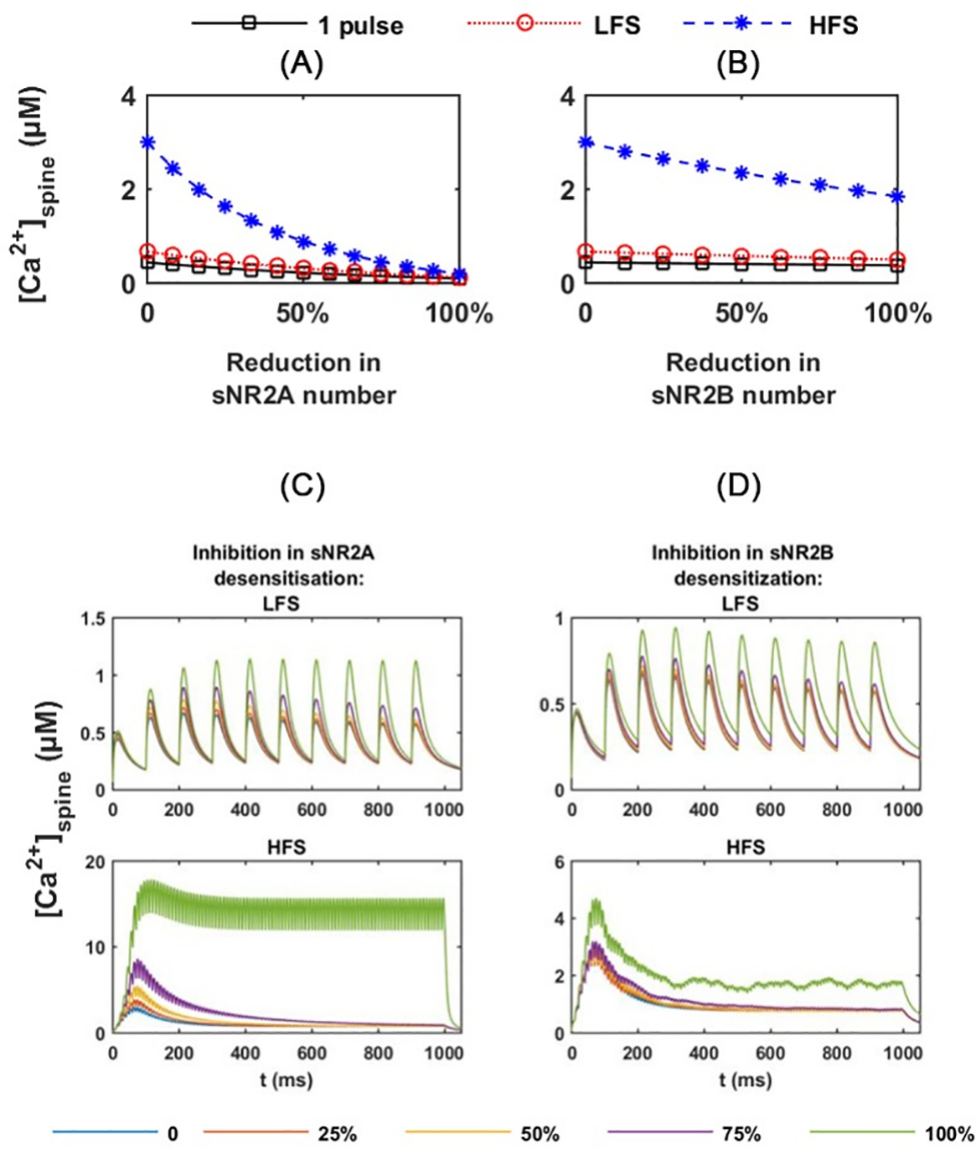
Effects of disturbance in synaptic NMDARs. (A) and (B) show effects of reductions in synaptic NR2A- and NR2B-NMDARs on the amplitudes of Ca^2+^ transient in the spine head, respectively. (C) and (D) show the Ca^2+^ time course in the spine head under LFS and HFS, with desensitisation inhibition of (C) sNR2A-NMDAR and (D) sNR2B-NMDAR, respectively. Percentages (%) denote the degree of reduction in the receptor numbers (A and B) and the degree of inhibition in receptor desensitisation (C and D). sNR2A: synaptic NR2A-NMDAR; sNR2B: synaptic NR2B-NMDAR.

The reduction in synaptic NR2A-NMDAR numbers shows negligible effects on [Ca^2+^]_peak_ under a 1 pulse stimulus and LFS, whereas, under HFS, the peak gradually decreases from 3 μM to nearly 0 μM (Fig 8A). The reduction in synaptic NR2B-NMDAR number only affects the [Ca^2+^]_peak_ in the spine head under HFS. There is about a 1 μM reduction in [Ca^2+^]_peak_ when the synaptic NR2B-NMDAR is fully removed (Fig 8B).

#### Effects of reductions in desensitisation of synaptic receptors

Under control conditions, NMDARs undergo desensitisation in response to the prolonged presence of their agonists, to prevent an excess Ca^2+^ influx [76]. A slow NMDA receptor desensitisation has been observed in transgenic mice that overexpress large amounts of Aβ within neurons [77, 78]. To mimic the inhibition of receptor desensitisation by AβO, we decrease the desensitisation related parameters (k_d1+_ and k_d2+_ in Table B in S2 Appendix) of each receptor by 0% to 100% of the standard.

The inhibition of desensitisation of synaptic NR2A-NMDAR and NR2B-NMDAR shows no obvious effects on Ca^2+^ transients in the spine head under a 1 pulse stimulus and LFS. Under HFS, [Ca^2+^]_peak_ increases significantly from 2.9 μM to 17.9 μM, with an increase in the inhibition level of synaptic NR2A-NMDAR desensitisation (Fig 8C). When desensitisation of the synaptic NR2A-NMDAR is fully inhibited, the Ca^2+^ concentration in the spine head maintains a high level after reaching a peak during the stimulation period. The inhibition of synaptic NR2B-NMDAR desensitisation also positively affect the Ca^2+^ transients in the spine head under HFS (Fig 8D). This leads to about a 2 μM increase in [Ca^2+^]_peak_ when the synaptic NR2B-NMDAR desensitisation is fully inhibited. However, the cytosolic Ca^2+^ concentration fails to maintain a high level after reaching a peak.

### Global sensitivity analysis

To identify the key players in AβO-induced Ca^2+^ dysregulation among the above factors, we apply global sensitivity analysis to Ca^2+^-Model. We select 15 factors that have been tested in previous sections (Table 2A) and eight outputs (Table 2B), which represent the postsynaptic neuron responses to presynaptic stimulation. We use a partial rank correlation coefficient (PRCC) to identify the most important or sensitive factors [79] (see Section 10 of S2 Appendix for details).

**Table 2:** Fifteen factors and eight outputs selected for PRCC. (A) Factors and their biological meaning, standard values and ranges for PRCC; (B) Outputs and their biological meaning for PRCC.

**A**			
**Factor**	**Biological meaning**	**Standard value**	**PRCC ranges**
G_0_	Glutamate number per release	1500	500–10000
[Glu]_rest_	Rest extrasynaptic glutamate concentration	0.25 uM	0–1 uM
[TP]	Glutamate transporter concentration	0.5 mM	0–1 uM
Ds2A	Inhibition on desensitisation of synaptic NR2A-NMDAR	0	0–1
Ds2B	Inhibition on desensitisation of synaptic NR2B-NMDAR		
DsAMPAR	Inhibition on desensitisation of synaptic AMPAR		
Dp2B	Inhibition on desensitisation of perisynaptic NR2B-NMDAR		
De2B	Inhibition on desensitisation of extrasynaptic NR2B-NMDAR		
DeAMPAR	Inhibition on desensitisation of extrasynaptic AMPAR		
#sNR2A	Synaptic NR2A-NMDAR number	12	6–18
#sNR2B	Synaptic NR2B-NMDAR number	8	4–12
#sAMPAR	Synaptic AMPAR number	85	43–130
#pNR2B	Perisynaptic NR2B-NMDAR number	3	1–5
#eNR2B	Extrasynaptic NR2B-NMDAR number	8	4–12
#eAMPAR	Extrasynaptic AMPAR number	20/μm^2^	15–30 /μm^2^
**B**			
**Output**	**Biological meaning**		
Ca^2+^ by sNR2A	Ca^2+^ ions enteringthrough synaptic NR2A-NMDAR		
Ca^2+^ by sNR2B	Ca^2+^ ions entering through synaptic NR2B-NMDAR		
Ca^2+^ by pNR2B	Ca^2+^ ions entering through perisynaptic NR2B-NMDAR		
Ca^2+^ by eNR2B	Ca^2+^ ions entering through extrasynaptic NR2B-NMDAR		
[Ca^2+^]_peak_ @ spine	Peak concentration of Ca^2+^ transient in spine head		
[Ca^2+^]_peak_ @ shaft	Peak concentration of Ca^2+^ transients in dendritic shaft		
V_peak_@spine	Peak membrane potential in spine head		
V_peak_@shaft	Peak membrane potential in dendritic shaft		

**Fig 9:**
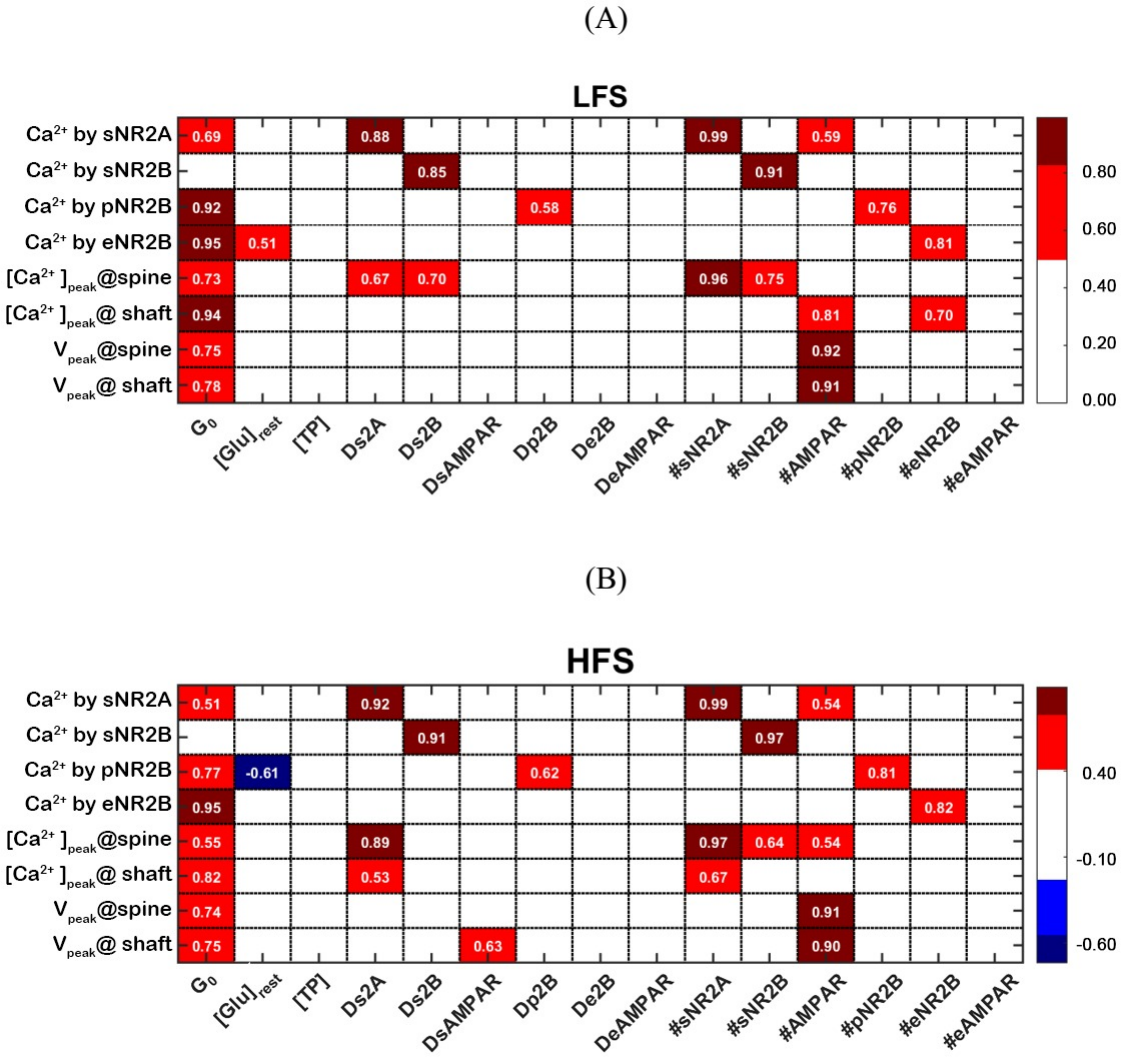
**Heat maps of PRCC results for the model in response to (A) LFS and (B) HFS.** The PRCC values for 15 factors against eight outputs are represented by colours, with the corresponding PRCC values written in white. Red and blue denote the positive and negative correlations, respectively. Only PRCCs greater than 0.5 and with a p-value < 0.05 are shown in the figures. The white colour means that there is no statistically significant relationship between the corresponding factor and the output.

Fig 9 shows that the number of glutamate molecules released after stimulation, G_0_, is the most important factor in synaptic transmission. G_0 _positively correlates with all outputs, except Ca^2+^ by sNR2A. The correlation levels decrease under HFS in comparison with under LFS. Moreover, [Glu]_rest_ negatively contributes to Ca^2+^ by pNR2B under HFS but not LFS, suggesting an elevation in [Glu]_rest_ will cause a stronger desensitisation of perisynaptic NR2B-NMDAR under HFS. [TP], De2B, DeAMPAR and #eAMPAR show no correlation with any output. The membrane depolarisation in the spine head and dendritic shaft are determined by G_0_ and #sAMPMR, but not by #eAMPAR. The results indicate that glutamate transporters and dendritic receptors are less involved in synaptic transmission than the synaptic receptors.

In the spine head, [Ca^2+^]_peak_ is the most sensitive to #sNR2A followed by #sNR2B. Under HFS, the correlation to #sNR2B decreases, which suggests a relatively lower activity of synaptic NR2B-NMDAR in HFS than under LFS. Ca^2+^ by sNR2A, but not Ca^2+^ by sNR2B, correlates with #sAMPAR, which infers that only synaptic NR2A-NMDAR is sensitive to membrane depolarisation. #sAMPAR becomes more important in HFS in comparison to LFS, indicating that a larger depolarisation by HFS brings more Ca^2+^ ions into the spine head.

In the dendritic shaft, [Ca^2+^]_peak_ is sensitive to #eNR2B under LFS and, surprisingly, it is also sensitive to #sNR2A under HFS. This suggests that under HFS increasing synaptic NR2A-NMDAR expression allows large numbers of Ca^2+^ ion influx and leads to more Ca^2+^ ions diffusing into the dendritic shaft.

### Effect of disturbances of synaptic NMDAR numbers on CaMKII state transition

Synaptic NMDARs are suggested to have a dual role in the formation of NMDAR-mediated-LTP by acting as Ca^2+^ channels (NR2A- and NR2B-NMDAR) and as a scaffold in PSD (NR2B-NMDAR) to anchor CaMKII [16, 17]. To further investigate how disturbances in synaptic NMDARs affect such a dual role, we integrate our Ca^2+^-Model with CaMKII-ST-Model by He et al.[44] (see Methods for model integration). We simulate the degree of reduction in the availability of a particular NMDAR type in PSD by decreasing the receptor number from the standard value (Table B in S2 Appendix) to zero. The stimulation protocols applied for the computational experiments are 1 s of pairing HFS [66] and 4 trains of pairing theta-burst stimulation (4 TBS), respectively, which are used for triggering LTP *in vivo* [80] (see Section 7 of S2 Appendix for a detailed explanation).

We investigate the Ca^2+^ elevation in the spine head and four chosen outputs: (1) numbers of Ca_4_CaM complexes; (2) numbers of autophosphorylated CaMKII subunits; (3) numbers of CaMKII in PSD; and (4) numbers of CaMKII-NMDAR complex in PSD. These outputs are the key factors from downstream events that determine the amount of CaMKII to be activated, autophosphorylated, translocated and anchored in PSD [81].

**Fig 10:**
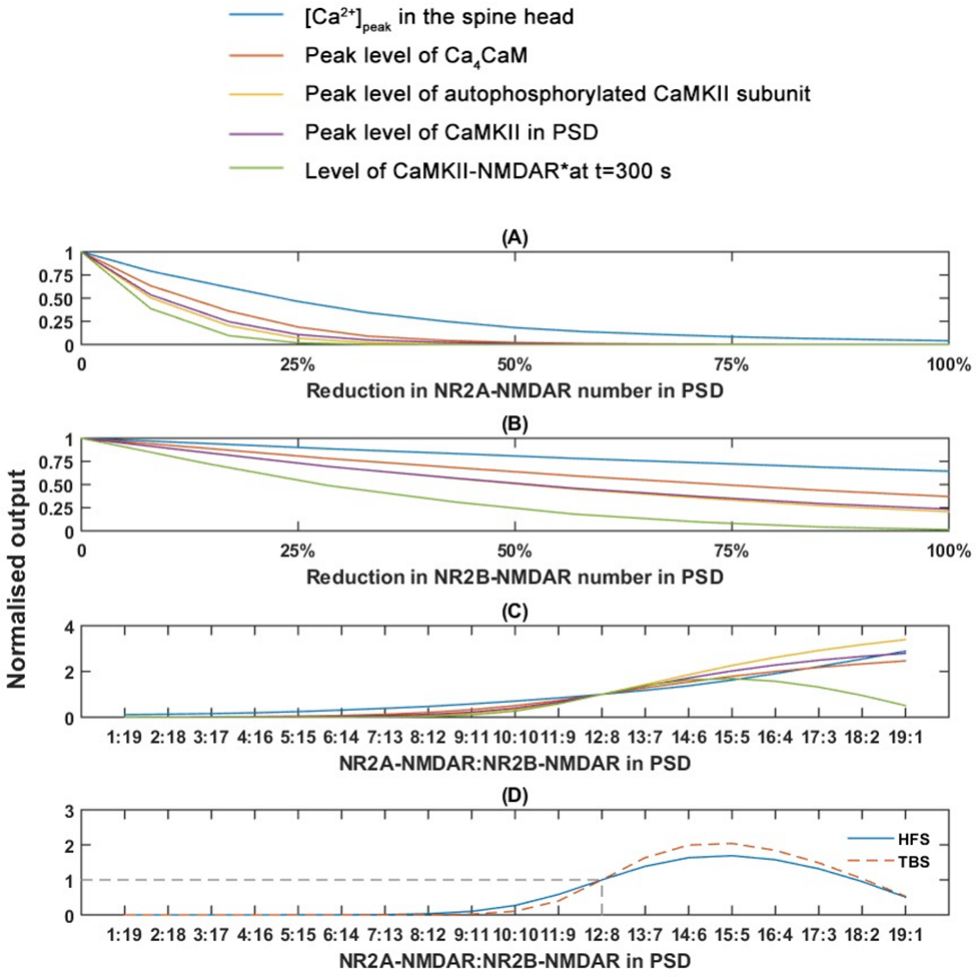
Effects of alterations in synaptic NMDAR numbers in response to pairing HFS. Effects of (A) reduction in synaptic NR2A-NMDAR number, (B) reduction in NR2B-NMDAR number and (C) alteration in the NR2A/NR2B ratio on selected typical outputs. (D) Effect of variation in the NR2A/NR2B ratio on the level of CaMKII-NMDAR complex production. The results are normalised to those under control condition (NR2A-NMDAR = 12, NR2B-NMDAR = 8; indicated by the dash lines).

The reduction in the numbers of NR2A-NMDAR in PSD greatly reduces [Ca^2+^]_peak_ in response to pairing HFS (Fig 10A) and 4 TBS (S3A Fig). The decrease in [Ca^2+^]_peak_ further reduces all four outputs. During both stimulation protocols, a 50% reduction (6 NR2A-NMDAR left in PSD) can block all downstream events (Fig 10A and S3A Fig). In particular, the formation of CaMKII-NMDAR is mostly sensitive to the NR2A-NMDAR reduction. Even an 8% reduction (11 NR2A-NMDAR left) can reduce the level of CaMKII-NMDAR formation by 60% (pairing HFS; Fig 10A) and 75% (4 TBS; S3A Fig), respectively. A 25% reduction (9 NR2A-NMDAR left in PSD) can lead to no production of CaMKII-NMDAR.

In contrast, a reduction in NR2B-NMDAR numbers in PSD affects the Ca^2+^ responses and all outputs less than the reduction in NR2A-NMDAR numbers (Fig 10B and S3B Fig). Even when reducing the NR2B-NMDAR from 8 to 1 (88% reduction), [Ca^2+^]_peak_ only decreases by about 35%. This decrease is not able to fully block most chosen downstream events, except for the formation of CaMKII-NMDAR complex. The formation of CaMKII-NMDAR complex is largely reduced by the reduction in NR2B-NMDAR numbers. A reduction of the NR2B-NMDAR numbers by 50% leads to over a 75% reduction in the final level CaMKII-NMDAR complex at t = 300 s.

Experimental evidence suggests that NR2A- and NR2B-NMDAR may contribute differently to the postsynaptic Ca^2+^ response and CaMKII activation. Both receptors are required in NMDAR-induced LTP [17]. The ratio of synaptic NR2A- to NR2B-NMDAR (NR2A/NR2B ratio) is precisely regulated by the production, trafficking and degradation of NMDARs. Different ratios regulate the preferences in the induction of different types of synaptic plasticity [82]. Therefore, we investigate the effects of disturbances on the NR2A/NR2B ratio in the CaMKII-NMDAR complex formation. We simulate the disturbance by varying the NR2A/NR2B ratio from 1: 19 to 19:1 while keeping the total number of NMDARs in PSD constant (20 NMDARs).

Fig 10C and S3C Fig show that [Ca^2+^]_peak_ stimulated by both pairing HFS and 4 TBS increases with the NR2A/NR2B ratio. The productions of the other four outputs are blocked when the NR2A/NR2B ratio is below 6:14. When a further increase in the NR2A/NR2B ratio occurs, the normalised peak levels of all outputs, except the production of CaMKII-NMDAR complex, rise with different slopes in response to a pairing HFS (Fig 10C and S3C Fig). In contrast, the normalised final levels of CaMKII-NMDAR after stimulation increase to maximum levels of 1.69 (pairing HFS) and 2.04 (pairing TBS), respectively, at the NR2A/NR2B ratio of 15:5, and decreases afterwards (Fig 10D). Therefore, our results indicate the existence of an optimal NR2A/NR2B ratio in the generation of the CaMKII-NMDAR complex.

## Discussion

In this work, we present computational models of Ca^2+^ dynamics in the dendritic spine and its parent dendrite shaft to investigate the effects of AβO-dependent disturbances on synaptic transmission. These disturbances are related to the availability of both glutamate and receptors. In particular, we include NMDARs with different subunit compositions and at different locations. Using simulations under different conditions, we study the activation patterns of specific NMDARs and the Ca^2+^ response at different locations.

Our results demonstrate that the increased glutamate release from the presynaptic terminal will promote Ca^2+^ responses mainly in the spine head. Global sensitivity analysis suggests a great sensitivity of the postsynaptic response to the number of glutamate molecules released during presynaptic stimulation. This confirms that the Aβ-induced increase in synaptic glutamate release plays a major role in the over-excitation and Ca^2+^ overload of postsynaptic neurons. The glutamate spillover from the synaptic cleft shows a much lower effect on the activation of extrasynaptic NMDARs than perisynaptic NMDARs. This is because of the fast diffusion of glutamate molecules and their uptake by glutamate transporters in astrocytes before they reach extrasynaptic NMDAR [47]. Therefore, Aβ-induced multiple vesicle releases from the presynaptic terminal alone are not sufficient to cause an overactivation of the extrasynaptic receptors.

Inhibition of glutamate uptake by glutamate transporters only affects the peri- and extrasynaptic receptors. On a longer time scale, this inhibition will cause a glutamate accumulation in the extrasynaptic space and lead to a gradual increase in [Glu]_rest_. We have shown that elevation in [Glu]_rest_ reduces the sensitivity of the postsynaptic neurons to the presynaptic signals, as a result of increased background opening of extrasynaptic NMDARs under the resting condition. Moreover, the AβO-induced astrocytic glutamate release also leads to a high-level Ca^2+^ ion influx in the absence of presynaptic stimulation. This finding is consistent with experimental observations [83], which show excitotoxicity results from Aβ-induced over-activation of the extrasynaptic NMDAR, but not the synaptic NMDAR. The overactivation can, in turn, promote Aβ production [84]. Even though these abnormal Ca^2+^ influxes have failed to induce large Ca^2+^ transients in the dendritic shaft, they still can potentially induce downstream pathways by affecting proteins located close to the receptors. Over a long time, this will contribute to the Ca^2+^ overload and neuronal death in AD [85]. Therefore, monitoring the extracellular glutamate concentration using precise measuring technology [86] could be useful for early diagnosis of AD, and therapeutic research can be carried out to investigate controlling the extracellular glutamate level to avoid excess activation of the extrasynaptic NMDARs.

Our simulation shows that synaptic NR2B-NMDAR contributes less to the synaptic Ca^2+^ transient compared to synaptic NR2A-NMDAR, in agreement with the simulation results from [87, 88]. Therefore, internalisation of synaptic NR2B-NMDAR disturbs the synaptic transmission [31, 39] not by affecting Ca^2+^ entry but, possibly, by disturbing interactions with other key players. Specifically, synaptic NR2B-NMDARs bind to CaMKII and are involved in mediating synapse strength and plasticity [89].

Simulation of the internalisation of synaptic NR2A- and NR2B-NMDAR shows negative effects on the activation of CaMKII and the formation of CaMKII-NMDAR complexes, to different degrees. Both types of NMDAR are necessary for LTP formation but they contribute to LTP induction and maintenance in different ways. Specifically, the role of NR2A-NMDARs is to allow a sufficient Ca^2+^ influx to trigger downstream Ca^2+^-CaM interactions, which determines the activation of CaMKII. NR2B-NMDAR contributes less as a Ca^2+^ channel than NR2A-NMDAR; however, it is required to function as a scaffold to anchor CaMKII in PSD. This result is consistent with the experimental findings and the hypothesis that the opening of NR2B-NMDAR is not necessary for LTP induction [23, 90]. Therefore, the internalisation of synaptic NR2B-NMDARs disturbs the synaptic functions without affecting the Ca^2+^ dynamics.

Simulation with different NR2A/NR2B ratios provides a clearer picture showing that NMDAR-LTP requires cooperation between the NR2A- and NR2B-NMDAR. AβO-induced internalisation of synaptic NMDAR in AD [31, 39] could underlie some of the critical alterations in the pathology of the disease. For instance, in AD transgenic mice, AβO has been observed to alter CaMKII distribution and reduce the synaptic CaMKII level [91]. Consequently, internalisation of synaptic NMDAR is suggested to contribute to the deficits of LTP and loss of synapses in AD [92, 93]. Therefore, selective inhibition on the internalisation of synaptic NR2B-NMDARs in AD (such as modifying key proteins in the NMDAR trafficking pathway [94]) could be a useful therapeutic approach that may prevent the loss of synapses and memory decline.

## Supporting information

S1 Fig Peak glutamate concentrations at different locations after stimulation. The simulation results are produced by various amounts of glutamate released in response to three types of stimulation patterns.
(TIF)

S2 Fig Ca^2+^ dynamics in the spine head and the dendritic shaft with the reduction in transporter levels. The glutamate number per release is 5000.
(TIF)

S3 Fig Effects of reduction in NR2A-NMDAR numbers in response to 4 TBS. Effects of (A) the reduction synaptic in NR2A-NMDAR numbers, (B) the reduction level in NR2A-NMDAR numbers and (C) the NR2A/NR2B ratio on selected typical outputs. The results are normalised to those under control condition (NR2A-NMDAR = 12, NR2B-NMDAR = 8).
(TIF)

S1 Appendix NMDAR. NMDAR and AβO-induced disturbances on the glutamatergic synaptic transmission.
(DOCX)

S2 Appendix Supporting information for the method section. (DOCX)
