# Supplementary figures and images for: Computational investigation of Amyloid-β-induced location- and subunit-specific disturbances of NMDAR at hippocampal dendritic spine in Alzheimer’s disease
Source: PLoS One. 2017 Aug 24;12(8):e0182743. doi: 10.1371/journal.pone.0182743 (PMC5570373; doi:10.1371/journal.pone.0182743)

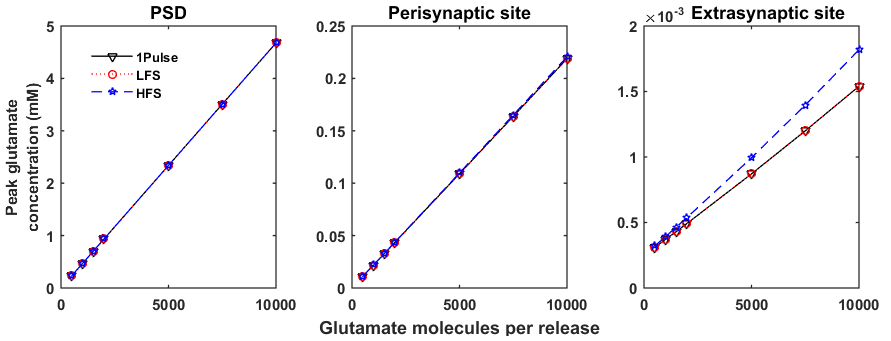

Supplement: S1 Fig — The simulation results are produced by various amounts of glutamate released in response to three types of stimulation patterns. (TIF) [file pone.0182743.s001.tif]

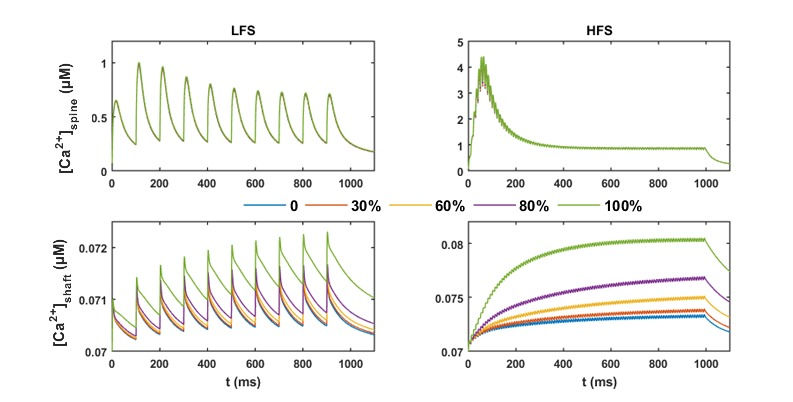

Supplement: S2 Fig — The glutamate number per release is 5000. (TIF) [file pone.0182743.s002.tif]

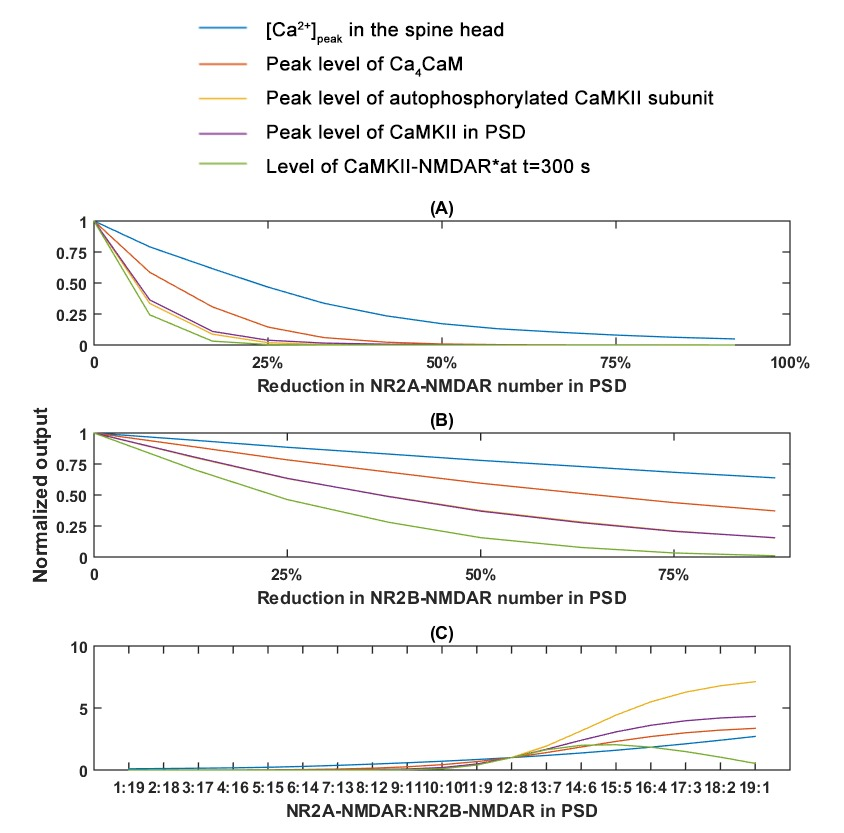

Supplement: S3 Fig — Effects of (A) the reduction synaptic in NR2A-NMDAR numbers, (B) the reduction level in NR2A-NMDAR numbers and (C) the NR2A/NR2B ratio on selected typical outputs. The results are normalised to those under control condition (NR2A-NMDAR = 12, NR2B-NMDAR = 8). (TIF) [file pone.0182743.s003.tif]
