# Supplementary material for: Computational investigation of Amyloid-β-induced location- and subunit-specific disturbances of NMDAR at hippocampal dendritic spine in Alzheimer’s disease
Source: PLoS One. 2017 Aug 24;12(8):e0182743. doi: 10.1371/journal.pone.0182743 (PMC5570373; doi:10.1371/journal.pone.0182743)
Supplement: S1 Appendix — NMDAR and AβO-induced disturbances on the glutamatergic synaptic transmission. (DOCX) [file pone.0182743.s004.docx]

## S1 Appendix. NMDAR

1. **NMDAR**

NMDAR is a heterotetramer, mostly comprising two NR1 and two NR2 subunits [1]. There are eight and four splice variants of NR1 and NR2 subunits, respectively. Those subunits share similar membrane topologies in that they contain an extracellular N-terminal domain with three transmembrane regions (M1, M3 and M4), a pore lining region (M2), an extracellular N-terminus and an intracellular C-terminus [2]. The agonists bind to their extracellular binding domains; in NR1 subunits it is the glycine-binding site, whereas, in NR2, it is the glutamate-binding sites. To activate NMDAR, all binding sites at the four subunits need to be occupied. Therefore, it requires the binding of two molecules of glutamate to the NR2 subunits and two molecules of agonist to the NR1 subunits. Recent research has found that NMDARs at different locations are gated by different co-agonists: D-serine for the synaptic NMDARs and glycine for the extrasynaptic NMDARs [3]. The affinity of NMDAR for glutamate depends on their NR2 subunit composition.

The NMDAR subunit compositions at different locations change during postnatal development [4]. The ratio of NR2A to NR2B increases at the synaptic site and decreases at the extrasynaptic site during postnatal development. In mature synapses, NR2A-NMDARs are predominant at the synaptic sites, which take about 60% of the total synaptic NMDARs [5]. In contrast, NMDARs located outside the synaptic region are mainly NR1/NR2B-NMDARs. They are proposed to play opposite physiological roles in mediating intracellular signalling and death pathways: activation of synaptic NMDARs shows neuroprotective effects, whereas stimulation of extrasynaptic NMDARs contributes to cell death [6].

In addition to the binding of glutamates and their co-agonists, Ca^2+^ entry through NMDAR requires the relief of Mg^2+^ blocks [7]. The Ca^2+^ permeation of NMDAR is mainly mediated by the M2 and M4 regions. The receptor is voltage-dependent and blocked by physiological concentrations of Mg^2+^ at resting membrane potential. During stimulation, the Mg^2+^ block is relieved by membrane depolarization, which can be produced by the opening of AMPARs. NMDARs are often found to be co-localized with AMPARs at the central synapses [8].

1. **AβO-induced disturbances on the glutamatergic synaptic transmission**

Experimental evidence suggests that AβO contributes to the upregulation in glutamate availability [9-11] by promoting glutamate release from presynaptic terminal [12-16] as well as potentiating the release of glutamate from astrocytes [17-19]. In addition, AβOs may disturb glutamate clearance mechanisms by reducing the expression levels of glutamate transporters or/and their activity [20-24]. These disturbances contribute to the increase in glutamate availability in the synaptic cleft and extrasynaptic space [10], which may consequently cause persistent activation of postsynaptic NMDAR and lead to receptor desensitisation and affect synaptic functions, such as synaptic plasticity (Li, Hong et al. 2009). Prolonged extrasynaptic NMDAR activation promotes neuronal AβO production (Bordji, Becerril-Ortega et al. 2010, Bordji, Becerril-Ortega et al. 2011). On the other hand, AβOs are reported to directly interact with NMDARs [25] and activate NR2B-NMDARs, leading to an increase in cytosolic Ca^2+^ levels [26]. AβOs may also reduce the number of NMDARs embedded in the plasma membrane at the synaptic location, without affecting its total expression [27-29]. In contrast, extrasynaptic NMDARs are not affected by the presence of AβOs [27]. Therefore, it is suggested that AβOs may play a role in mediating the trafficking of NMDARs, especially by promoting synaptic NR2B-NMDAR endocytosis. The internalised NMDARs will lose their function as membrane Ca^2+^ channels and cannot anchor CaMKII in PSD.

### Reference

1. Furukawa H, Singh SK, Mancusso R, Gouaux E. Subunit arrangement and function in NMDA receptors. Nature. 2005;438(7065):185-92. doi: <http://www.nature.com/nature/journal/v438/n7065/suppinfo/nature04089_S1.html>.

2. Cull-Candy SG. NMDA Receptors. eLS: John Wiley & Sons, Ltd; 2001.

3. Papouin T, Ladépêche L, Ruel J, Sacchi S, Labasque M, Hanini M, et al. Synaptic and extrasynaptic NMDA receptors are gated by different endogenous coagonists. Cell. 2012;150(3):633-46.

4. Petralia RS. Distribution of Extrasynaptic NMDA Receptors on Neurons. The Scientific World Journal. 2012;2012:11. doi: 10.1100/2012/267120.

5. Cheng D, Hoogenraad CC, Rush J, Ramm E, Schlager MA, Duong DM, et al. Relative and Absolute Quantification of Postsynaptic Density Proteome Isolated from Rat Forebrain and Cerebellum. Molecular & Cellular Proteomics. 2006;5(6):1158-70. doi: 10.1074/mcp.D500009-MCP200.

6. Hardingham GE, Bading H. Synaptic versus extrasynaptic NMDA receptor signalling: implications for neurodegenerative disorders. Nature reviews Neuroscience. 2010;11(10):682-96. Epub 2010/09/16. doi: 10.1038/nrn2911. PubMed PMID: 20842175; PubMed Central PMCID: PMCPmc2948541.

7. Mayer ML, Westbrook GL, Guthrie PB. Voltage-dependent block by Mg2&plus; of NMDA responses in spinal cord neurones. 1984.

8. Dingledine R, Borges K, Bowie D, Traynelis SF. The Glutamate Receptor Ion Channels. Pharmacological Reviews. 1999;51(1):7-62.

9. Ondrejcak T, Klyubin I, Hu NW, Barry AE, Cullen WK, Rowan MJ. Alzheimer's disease amyloid beta-protein and synaptic function. Neuromolecular medicine. 2010;12(1):13-26. Epub 2009/09/17. doi: 10.1007/s12017-009-8091-0. PubMed PMID: 19757208.

10. Danysz W, Parsons CG. Alzheimer's disease, beta-amyloid, glutamate, NMDA receptors and memantine--searching for the connections. Br J Pharmacol. 2012;167(2):324-52. Epub 2012/06/01. doi: 10.1111/j.1476-5381.2012.02057.x. PubMed PMID: 22646481; PubMed Central PMCID: PMCPmc3481041.

11. Butterfield DA, Pocernich CB. The glutamatergic system and Alzheimer’s disease. CNS drugs. 2003;17(9):641-52.

12. Bobich JA, Zheng Q, Campbell A. Incubation of nerve endings with a physiological concentration of Aβ_ {1-42} activates CaV2. 2 (N-Type)-voltage operated calcium channels and acutely increases glutamate and noradrenaline release. Journal of Alzheimer's Disease. 2004;6(3):243-55.

13. Kabogo D, Rauw G, Amritraj A, Baker G, Kar S. β-amyloid-related peptides potentiate K+-evoked glutamate release from adult rat hippocampal slices. Neurobiology of aging. 2010;31(7):1164-72.

14. Chin JH, Ma L, MacTavish D, Jhamandas JH. Amyloid β protein modulates glutamate-mediated neurotransmission in the rat basal forebrain: involvement of presynaptic neuronal nicotinic acetylcholine and metabotropic glutamate receptors. The Journal of Neuroscience. 2007;27(35):9262-9.

15. Abramov E, Dolev I, Fogel H, Ciccotosto GD, Ruff E, Slutsky I. Amyloid-β as a positive endogenous regulator of release probability at hippocampal synapses. Nature neuroscience. 2009;12(12):1567-76.

16. Arias C, Arrieta I, Tapia R. β‐Amyloid peptide fragment 25–35 potentiates the calcium‐dependent release of excitatory amino acids from depolarized hippocampal slices. Journal of neuroscience research. 1995;41(4):561-6.

17. Noda M, Nakanishi H, Akaike N. Glutamate release from microglia via glutamate transporter is enhanced by amyloid-beta peptide. Neuroscience. 1999;92(4):1465-74.

18. Orellana JA, Shoji KF, Abudara V, Ezan P, Amigou E, Sáez PJ, et al. Amyloid β-induced death in neurons involves glial and neuronal hemichannels. The Journal of Neuroscience. 2011;31(13):4962-77.

19. Kuchibhotla KV, Lattarulo CR, Hyman BT, Bacskai BJ. Synchronous hyperactivity and intercellular calcium waves in astrocytes in Alzheimer mice. Science (New York, NY). 2009;323(5918):1211-5. Epub 2009/03/03. doi: 10.1126/science.1169096. PubMed PMID: 19251629; PubMed Central PMCID: PMCPmc2884172.

20. Harris ME, Carney JM, Cole PS, Hensley K, Howard BJ, Martin L, et al. beta-Amyloid peptide-derived, oxygen-dependent free radicals inhibit glutamate uptake in cultured astrocytes: implications for Alzheimer's disease. Neuroreport. 1995;6(14):1875-9. PubMed PMID: 8547588.

21. Harris ME, Wang Y, Pedigo NW, Hensley K, Butterfield DA, Carney JM. Amyloid β Peptide (25–35) Inhibits Na+‐Dependent Glutamate Uptake in Rat Hippocampal Astrocyte Cultures. Journal of neurochemistry. 1996;67(1):277-86.

22. Parpura-Gill A, Beitz D, Uemura E. The inhibitory effects of β-amyloid on glutamate and glucose uptakes by cultured astrocytes. Brain Research. 1997;754(1–2):65-71. doi: <http://dx.doi.org/10.1016/S0006-8993(97)00043-7>.

23. Matos M, Augusto E, Oliveira C, Agostinho P. Amyloid-beta peptide decreases glutamate uptake in cultured astrocytes: involvement of oxidative stress and mitogen-activated protein kinase cascades. Neuroscience. 2008;156(4):898-910.

24. Fernández-Tomé P, Brera B, Arévalo Ma-A, de Ceballos MaL. β-Amyloid 25-35 inhibits glutamate uptake in cultured neurons and astrocytes: modulation of uptake as a survival mechanism. Neurobiology of disease. 2004;15(3):580-9.

25. De Felice FG, Velasco PT, Lambert MP, Viola K, Fernandez SJ, Ferreira ST, et al. Aβ oligomers induce neuronal oxidative stress through an N-methyl-D-aspartate receptor-dependent mechanism that is blocked by the Alzheimer drug memantine. Journal of Biological Chemistry. 2007;282(15):11590-601.

26. Ferreira IL, Bajouco LM, Mota SI, Auberson YP, Oliveira CR, Rego AC. Amyloid beta peptide 1–42 disturbs intracellular calcium homeostasis through activation of GluN2B-containing N-methyl-d-aspartate receptors in cortical cultures. Cell Calcium. 2012;51(2):95-106. doi: <http://dx.doi.org/10.1016/j.ceca.2011.11.008>.

27. Snyder EM, Nong Y, Almeida CG, Paul S, Moran T, Choi EY, et al. Regulation of NMDA receptor trafficking by amyloid-beta. Nat Neurosci. 2005;8(8):1051-8. Epub 2005/07/19. doi: 10.1038/nn1503. PubMed PMID: 16025111.

28. Goto Y, Niidome T, Akaike A, Kihara T, Sugimoto H. Amyloid β-peptide preconditioning reduces glutamate-induced neurotoxicity by promoting endocytosis of NMDA receptor. Biochemical and Biophysical Research Communications. 2006;351(1):259-65. doi: <http://dx.doi.org/10.1016/j.bbrc.2006.10.030>.

29. Lacor PN, Buniel MC, Furlow PW, Clemente AS, Velasco PT, Wood M, et al. Aβ oligomer-induced aberrations in synapse composition, shape, and density provide a molecular basis for loss of connectivity in Alzheimer's disease. The Journal of Neuroscience. 2007;27(4):796-807.
