# Supplementary material for: Computational investigation of Amyloid-β-induced location- and subunit-specific disturbances of NMDAR at hippocampal dendritic spine in Alzheimer’s disease
Source: PLoS One. 2017 Aug 24;12(8):e0182743. doi: 10.1371/journal.pone.0182743 (PMC5570373; doi:10.1371/journal.pone.0182743)
Supplement: S2 Appendix — (DOCX) [file pone.0182743.s005.docx]

## S2 Appendix. Supporting information for the method section

### SubModel 1: Presynaptic glutamate release, extrasynaptic diffusion space definition and glutamate uptake by transporters

A schematic of SubModel 1 in two-dimensions is given in Figure A.


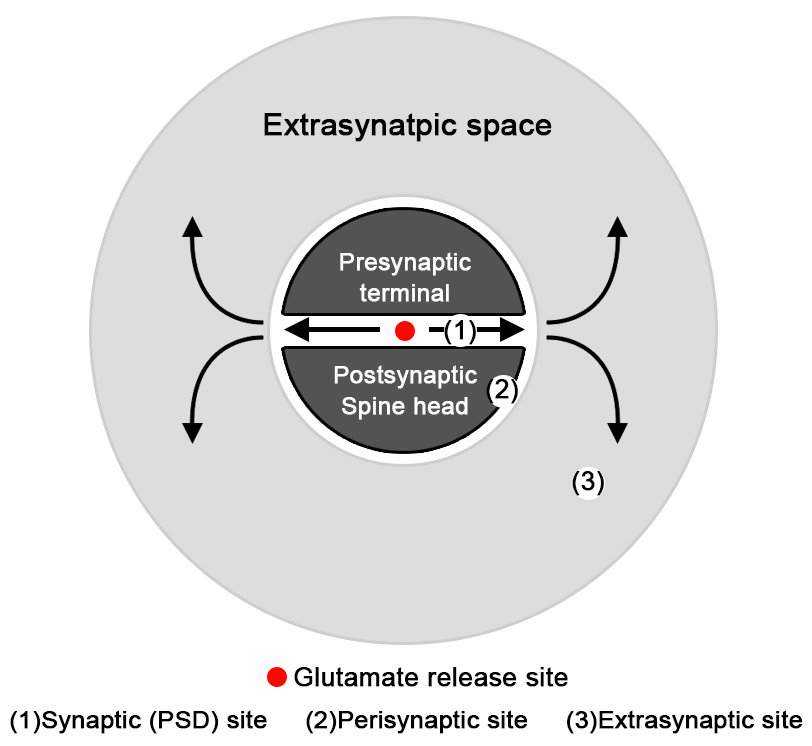


Figure A. Schematic two-dimensional profile of SubModel 1. Glutamate is released from the centre of synaptic cleft and diffused across the synaptic cleft and into the extrasynaptic space. The synaptic cleft is assumed to be a flat cylinder with 20 thin concentric shells. The extrasynaptic space is a porous medium with spherical concentric shells. The arrows denote the diffusion direction of glutamate. Glutamate transporters are homogenously distributed in the extrasynaptic space. Three grey areas (marked with (1), (2) and (3)) represent the synaptic (PSD), perisynaptic and extrasynaptic sites. The glutamate concentration at each site is used to calculate local receptor activity in the NMDAR and AMPAR models. Based on the model geometry data in Table A, the extrasynaptic site is about 826 nm away from the release point.

#### Presynaptic glutamate release

The glutamate vesicle is assumed to be released from a point site in the centre of the presynaptic terminal surface. The time course of a single glutamate vesicle release is modelled by the function

|  | $\phi\left( t \right)= \sigma^{2}t\exp\left( -\sigma t \right) ,$ | (1) |
| --- | --- | --- |

where $\sigma$ (= 39 ms^-1^ ) is the release time constant [1].

#### Extrasynaptic diffusion space define

The spherical extrasynaptic space is modelled as 50 concentric shells with a thickness of 20 nm. The resting level of glutamate concentration in the extrasynaptic space is set to 0.25 μM [2, 3]. The glutamate concentration in the last cylinder shell, which is 1.36 μm from the release site, is fixed at resting level (open boundary condition).

#### Glutamate uptake by transporters

A gap between the glial sheath and spine head is assumed and, thus, the distribution of glutamate transporters starts at 20 nm from the edge of the dendritic cleft. A simple kinetic scheme is applied to the glutamate binding and uptake by glial transporters

|  | $Glu+Tr\underset{\leftrightarrow}{k_{1f}; k_{1b}}Glu-Tr\underset{\to}{k_{2}}Tr ,$ |  |
| --- | --- | --- |

where $Glu$is the glutamate, $Tr$ is the unbound surface transporter and $Glu-Tr$ is the glutamate-transporter complex [1]. The values of the reaction constants, $k_{1f}$, $k_{1b}$ and $k_{2}$ are listed in Table A.

Table A. Model geometry and glutamate transmission related parameters.

| **Description** | **Symbol** | **Value** | **Reference** |
| --- | --- | --- | --- |
| Volume of spine head |  | 0.1 μm^3^ |  |
| Volume of PSD |  | 0.01 μm^3^ |  |
| Radius of PSD | $r_{PSD}$ | 150 nm | [1, 4] |
| Height of cleft | $h_{cleft}$ | 20 nm |  |
| Radius of cleft | $r_{cleft}$ | 363 nm |  |
| Length of dendritic neck | $l_{neck}$ | 750 nm | [5, 6] |
| Radius of dendritic neck | $r_{neck}$ | 50 nm |  |
| Length of dendritic shaft | $l_{shaft}$ | 1000 nm | [5, 7] |
| Radius of dendritic shaft | $r_{shaft}$ | 500 nm |  |
| Distance of the glial sheath from the synaptic cylinder surface |  | 20 nm | [8] |
| Thickness of cylinder shell (cleft) | ${dr}_{1}$ | 10 nm |  |
| Thickness of sphere shell (extrasynaptic space) | ${dr}_{2}$ | 20 nm |  |
| Glutamate vesicle content | ${Glu}_{0}$ | 1500 molecules | [9] |
| Glutamate diffusion constant in the cleft | $D_{Glu}$ | 0.2 μm^2^ ms^-1^ | [1]  (20-24 °C) |
| Tortuosity factor | $\lambda$ | 1.34 |  |
| Extracellular volume fraction | $\alpha$ | 0.12 |  |
| Transporter concentration | $B_{total}$ | 0.5 mM |  |
| Binding rate constant | $k_{1f}$ | 0.1 ms^-1^ |  |
| Unbinding rate constant | $k_{1b}$ | 5 mM^-1^ ms^-1^ |  |
| Translocation rate | $k_{2}$ | 0.1 ms^-1^ |  |
| Resting glutamate concentration |  | 0.25 μM | [2, 3] |

### Markov kinetic scheme of NMDAR and AMPAR and parameters


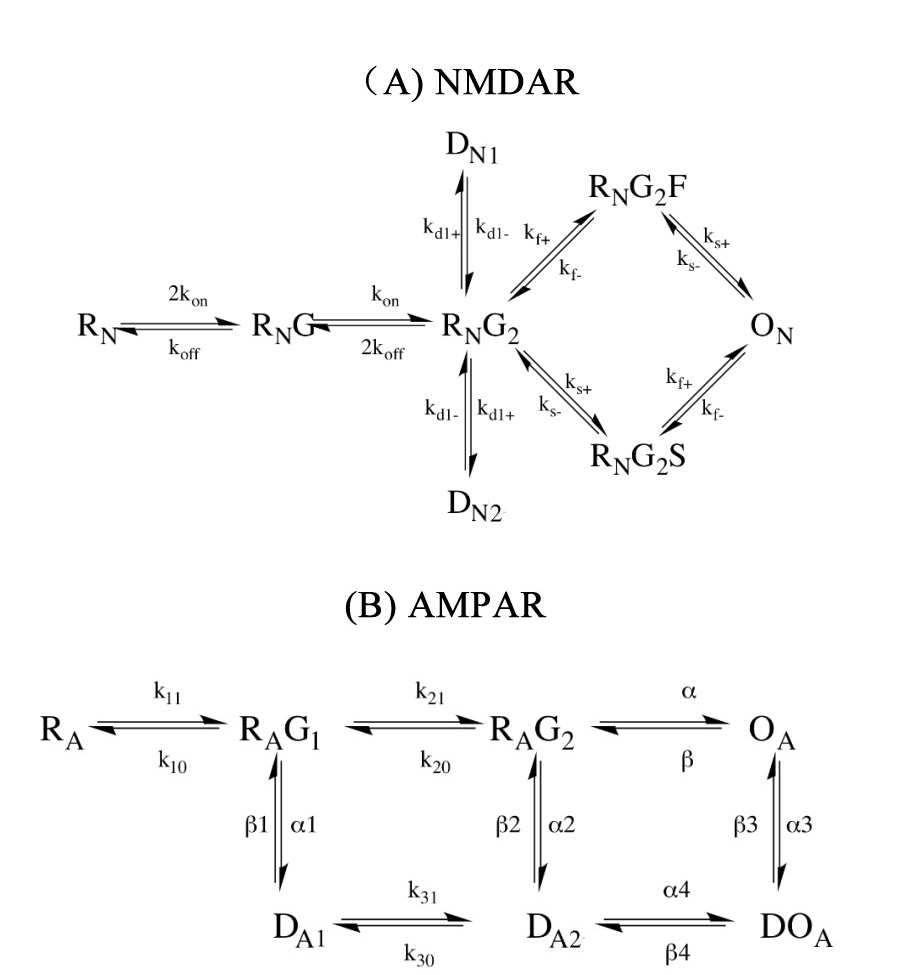


**Figure B.** **Markov kinetic scheme of (A) NMDAR and (B) AMPAR.** (A) The state transition of NMDAR is simulated by using a subtype-specific kinetic model [10], which assumes that the two NR1 subunits are always occupied by coagonists. Therefore, the activation of the receptor depends only on the glutamate concentration. NR1/NR2A-NMDAR and NR1/NR2B-NMDAR share the same activation schemes but with different reaction rate constants (Table B in S2 Appendix), which allows us to investigate the effects of the different types of NMDAR. This eight-state kinetic scheme incorporates separate binding steps of two glutamate molecules (R_N_G and R_N_G_2_), two desensitised states (D_N1_ and D _N2_) and two conformational change stages (R_N_G_2_F and R_N_G_2_S) before opening (O_N_). Channel opening requires both faster and slower conformational changes in NR1 and NR2 subunits, respectively. These two transition processes are independent and can happen in any order. (B) The dynamics of a single AMPAR is simulated by a seven-state model [11], which contains binding states of one and two glutamate molecules (R_A_G_1_ and R_A_G_2_), the open state (O_A_), and their corresponding desensitised states (D_A1_, D_A2_, DO_A_). The kinetic rate constants are listed in Table B.

Table B. NMDAR and AMPAR parameters.

| **Receptor type** | **Location** | **Number** | **Reference** |
| --- | --- | --- | --- |
| NR2A–NMDAR | PSD (synaptic NR2A-NMDAR) | 12 | [12-14] |
|  | Perisynaptic site | 0 |  |
|  | Extrasynaptic site | 0 |  |
| NR1/NR2B-NMDAR | PSD  (synaptic NR2B-NMDAR) | 8 |  |
|  | Perisynaptic site (perisynaptic NR2B-NMDAR) | 3 |  |
|  | Extrasynaptic site (Extrasynaptic NR2b-NMDAR) | 8 |  |
| AMPAR | PSD (synaptic AMPAR) | 85 receptors |  |
|  | Extrasynaptic site (extrasynaptic AMPAR) | 20 receptors/μm2 | [15] |
| **Receptor type** | **Reaction rate constants** | **Value** | **Reference** |
| NR2A–NMDARs  (20-24 °C) | $k_{\mathrm{on}}$ | 0.0316 µM^-1^ ms^-1^ | [10] |
|  | $k_{\mathrm{off}}$ | 1.01 ms^-1^ |  |
|  | $k_{d1+}$ | 0.0851 ms^-1^ |  |
|  | $k_{d1-}$ | 0.0297 ms^-1^ |  |
|  | $k_{d2+}$ | 0.23 ms^-1^ |  |
|  | $k_{d2-}$ | 0.00101 ms^-1^ |  |
|  | $k_{f+}$ | 0.230 ms^-1^ |  |
|  | $k_{f-}$ | 0.178 ms^-1^ |  |
|  | $k_{s+}$ | 3.140 ms^-1^ |  |
|  | $k_{s-}$ | 0.174 ms^-1^ |  |
| NR2B–NMDARs  (20-24 °C) | $k_{\mathrm{on}}$ | 0.00283 µM^-1^ ms^-1^ |  |
|  | $k_{\mathrm{off}}$ | 0.0381 ms^-1^ |  |
|  | $k_{d1+}$ | 0.550 ms^-1^ |  |
|  | $k_{d1-}$ | 0.0814 ms^-1^ |  |
|  | $k_{d2+}$ | 0.112 ms^-1^ |  |
|  | $k_{d2-}$ | 0.00091ms^-1^ |  |
|  | $k_{f+}$ | 0.048 ms^-1^ |  |
|  | $k_{f-}$ | 0.23 ms^-1^ |  |
|  | $k_{s+}$ | 2.836 ms^-1^ |  |
|  | $k_{s-}$ | 0.175 ms^-1^ |  |
| AMPAR  (22 °C) | $k_{11}$ | 0.00459 µM^-1^ ms^-1^ | [11] |
|  | $k_{10}$ | 4.26 ms^-1^ |  |
|  | $k_{21}$ | 0.0284 µM^-1^ ms^-1^ |  |
|  | $k_{20}$ | 3.26 ms^-1^ |  |
|  | $k_{31}$ | 0.00127 µM^-1^ ms^-1^ |  |
|  | $k_{30}$ | 0.0457 ms^-1^ |  |
|  | $\alpha$ | 4.24 ms^-1^ |  |
|  | $\beta$ | 0.9 ms^-1^ |  |
|  | $\alpha_{1}$ | 2.89 ms^-1^ |  |
|  | $\beta_{1}$ | 0.0392 ms^-1^ |  |
|  | $\alpha_{2}$ | 0.172 ms^-1^ |  |
|  | $\beta_{2}$ | 0.000727 ms^-1^ |  |
|  | $\alpha_{3}$ | 0.0177 ms^-1^ |  |
|  | $\beta_{3}$ | 0.004 ms^-1^ |  |
|  | $\alpha_{4}$ | 0.0168 ms^-1^ |  |
|  | $\beta_{4}$ | 0.1904 |  |

### Membrane potential

We construct the electrical model (Figure C) based on the morphological features described in the Method section.


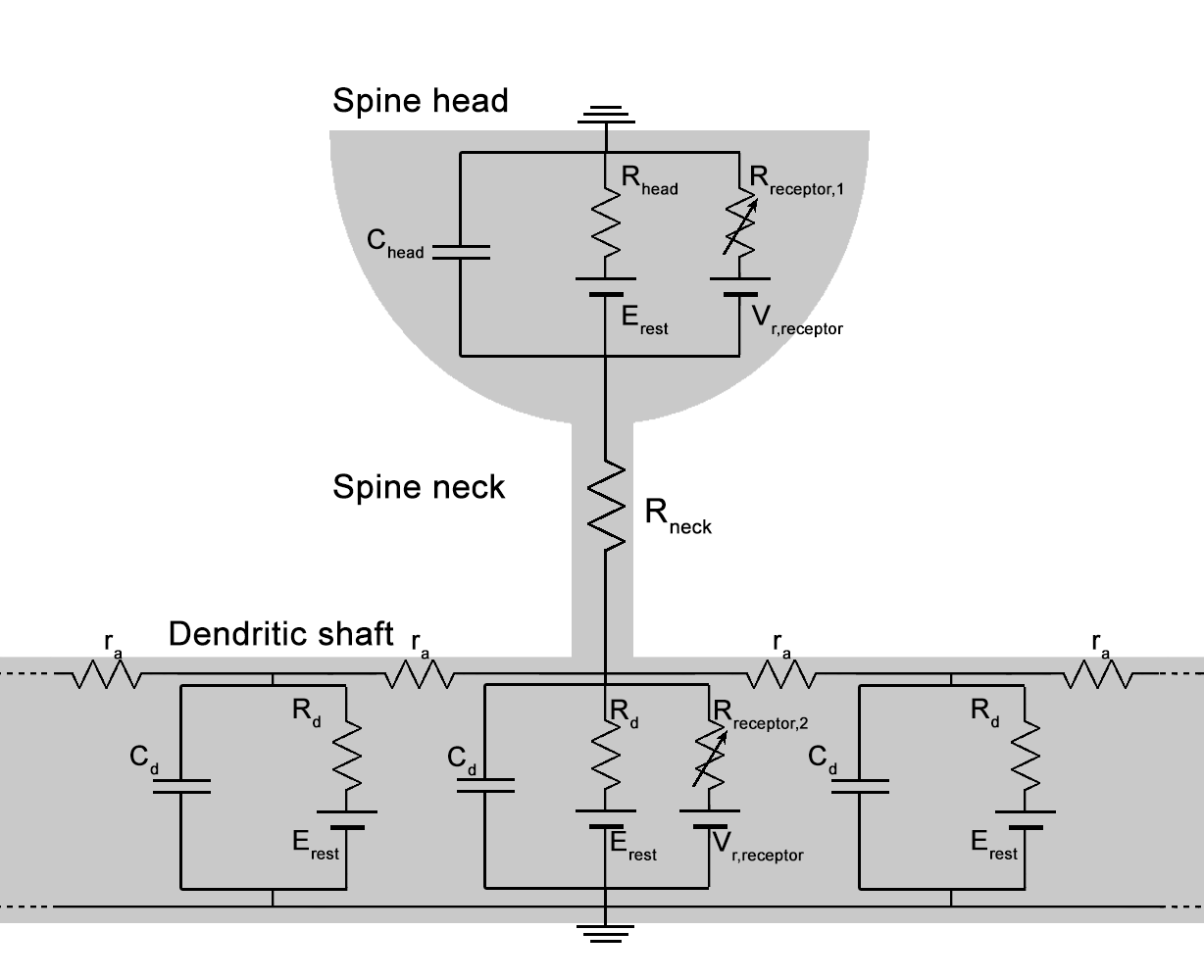


Figure C. Passive electrical model of a dendritic spine and the adjacent dendritic shaft of a CA1 pyramidal neuron. The spine head and dendritic shaft are modelled as separate compartments, with membrane capacitances, C_head_ and C_d_, and resistances, Rhead and Rd, respectively. R_receptor,1_ and R_receptor,2_ are the resistances of receptors in the membrane of the spine head and the dendritic shaft, respectively. The spine head and dendritic compartments are connected by the spine neck, with neck resistance, R_neck_. The dendritic shaft is modelled as a series of identical cylindrical compartments. The resting potential, E_rest_, is assumed to be the same in all compartments (E_rest_ =-70 mV).

The spine head is modelled as an isopotential hemispherical compartment. Its membrane resistance and capacitance can be calculated by its surface area ($A_{head}$), the specific membrane resistance ($R_{m}$) and the specific capacitance ($C_{m})$[16]: $R_{\mathrm{head}}=\frac{R_{m}}{A_{head}}$ and $C_{head}= C_{m}A_{head}$. Therefore, the postsynaptic membrane potential is

|  | $C_{head}\frac{dV_{head}}{dx}=\frac{E_{rest}-V_{0}}{R_{head}}-I_{syn}+I_{receptor,1} ,$ | (2) |
| --- | --- | --- |

where $I_{receptor,1}$ represents the current of receptors on the spine head and $I_{syn}$ represents the current flow through the spine neck. Based on Ohm’s law, $I_{syn}$ can be calculated as the voltage drop from the spine head ($V_{head}$) to the dendrite shaft ($V_{d}$) across the spine neck resistance ($R_{neck}$)

$$I_{syn}=\frac{V_{head}-V_{d}}{R_{neck}} .$$

The spine neck is simulated as an electrical cylindrical resistor of length $l_{neck}$ and radius$r_{neck}$, therefore, its resistance is

$$R_{neck}=R_{i}l_{neck}\pi r_{neck}^{2} ,$$

where $R_{i}$ is the specific axial resistance (or specific cytoplasmic resistivity). In the CA1 pyramidal neuron, the values of neck length and width are taken as their average values from the physiological ranges [6].

The basal dendrite is represented as a series of *N* cylindrical compartments with sealed ends on both sides [17]. Each compartment has a length of $\lambda$ and a diameter of$d$. The sealed end boundary condition assumes that the resistance of the compartment is very high; therefore, the exit current flow via the end is negligible. We assume that only one spine is attached to the middle point of the dendrite. Therefore, for compartment *j* out of *N* dendritic compartments, the change in its membrane potential ($V_{d,n}$) is given by the following differential equations:

| $\tau_{m}\frac{dV_{d,1}}{dt}=E_{rest}-V_{d,1}+2V_{d,2}-2V_{d,1}+\sqrt{\frac{4R_{m}R_{i}}{\pi^{2}d^{3}}}\left( I_{syn}+I_{receptor,2} \right) ,$ | (3) |
| --- | --- |
| $\tau_{m}\frac{dV_{d,n}}{dt}=E_{rest}-V_{d,n}+\left( V_{d,n+1}-2V_{d,n}+V_{d,n} \right), n=2\ldots N-1 , and$ | (4) |
| $\tau_{m}\frac{dV_{d,N}}{dt}=E_{rest}-V_{d,1}+2V_{d,N-1}-2V_{d,N}$. | (5) |

The membrane potential related parameters are listed in Table C.

Table C. Membrane potential related parameters.

| **Description** | **Symbol** | **Value** | **Reference** |
| --- | --- | --- | --- |
| Specific resistance | $R_{m}$ | 30 kΩ∙ cm^2^ | [18] |
| Specific capacitance | $C_{m}$ | 1 μF/cm^2^ |  |
| Specific cytoplasmic resistivity | $R_{i}$ | 100 kΩ∙ cm |  |
| Membrane time constant | $\tau_{E}$ | $R_{m}{\cdot C}_{m}$ |  |
| Resting membrane potential | $E_{\mathrm{rest}}$ | -70 mV | [19] |
| Single-channel conductance of NMDAR | $g_{\mathrm{NMDAR}}$ | 45 pS (20-24 °C) | [20] |
| Single-channel conductance of AMPAR | $g_{\mathrm{AMPAR}}$ | 10 pS (20-24 °C) | [20] |
| Reversal potentials for NMDAR and AMPAR | $V_{r,NMDAR}$  $V_{r,AMPAR}$ | 0 mV | [21] |
| Extracellular magnesium concentration | $[Mg]$ | 1 mM | [21] |

The total input currents of the spine head and the shaft are a summation of the current of both local NMDARs and AMPARs:

$I_{receptor,1}=I_{NMDAR,PSD}+I_{NMDAR,perisynaptic}+I_{AMPAR,PSD} ,$ and

$I_{receptor,2}=I_{NMDAR,d}+I_{AMPAR,d}$ .

The receptor currents at a specific location $i$ ($I_{NMDAR,i}$ and $I_{AMPAR,i}$) are

|  | $I_{NMDAR,i}= g_{NMDAR} N_{NMDAR,i}{Po}_{NMDAR,i}B\left( V_{i} \right)\left( V_{i}-V_{r,NMDAR} \right)$ , and | (6) |
| --- | --- | --- |
|  | $I_{AMPAR,i}=g_{AMPAR}N_{AMPAR,i}{Po}_{AMPAR,i} \left( V_{i}-V_{r,AMPAR} \right) ,$ | (7) |

where $V_{i}$ is the membrane voltage of site $i$ ($i$=1, postsynaptic density (PSD); 2, perisynaptic site; 3, extrasynaptic site), $V_{r,NMDAR}$and $V_{r,AMPAR}$ are the reversal potentials for NMDAR and AMPAR, respectively. $g_{NMDAR}$ and $g_{AMPAR}$ are the single channel conductances of NMDARs and AMPARs, respectively$.N_{NMDAR,i}$and $N_{AMPAR,i}$ are the total NMDAR and AMPAR numbers at site $i$, respectively.

Eq.(6) describes the Mg^2+^ blockage of NMDARs ($B\left( V_{i} \right)$) under $V_{i}$ at each time step [21]

|  | $B\left( V_{i} \right)=\frac{1}{1+\frac{\left[ Mg \right]}{3.57}\exp\left( -0.062V_{i} \right)}$ ， | (8) |
| --- | --- | --- |

where $[Mg]$ is the extracellular magnesium concentration (1 mM).

### SubModel 3: Compartmental model of Ca^2+^ flux in a dendritic spine

The schematic diagram of SubModel 3 is in Figure D. Extracellular Ca^2+^ions entering each compartment result from the local membrane Ca^2+^current that is then buffered by various Ca^2+^ buffer proteins extruded through membrane Ca^2+^ pumps and diffused from the spine head to the dendrite. We assume that the molecules are well-mixed in each compartment and only diffusion of Ca^2+^ between the compartments is considered. The Ca^2+^ dynamics in PSD, spine head, spine neck and dendritic shaft are governed by

|  | $\frac{d\left[ {Ca}^{2+} \right]_{j}}{dt}=J_{I_{Ca},j}+J_{mem,j}+J_{diffusion,j}-J_{buffer,j} ,$ | (9) |
| --- | --- | --- |

where $j$ indicates the compartment index ($j$=1, PSD; 2, cytosol; 3, spine neck; 4, dendritic shaft). The values of the parameters are given in Table D.





Figure D. Schematic diagram of SubModel 3: Ca^2+^ dynamics at the dendritic spine head and its adjacent dendritic shaft. The spine head is divided into two compartments, PSD and cytosol. A thin long spine neck links the spine head to the dendritic shaft, which allows Ca^2+^ to diffuse from the spine head to the dendrite. Ca^2+^ enters PSD, cytosol and the dendritic shaft through NMDARs and is extruded by Ca^2+^ pumps at all compartments. Ca^2+^ buffers are distributed homogenously within each compartment. Ca^2+^ diffusion between adjacent compartments is also considered. This schematic does not represent the actual scale of the geometry of SubModel 3.

Table D. Parameters for the spine compartment model.

| **Description** | **Symbol** | **Value** | **Reference** |
| --- | --- | --- | --- |
| Pump affinity (PMCA) | $K_{M,1}$ | 0.2 μΜ | [22]  [23, 24] |
| Turnover rate (PMCA) | $V_{max,1}$ | 100 ms^-1^(37 °C) |  |
| Pump affinity (NCX) | $K_{M,2}$ | 20 Μm |  |
| Turnover rate (NCX) | $V_{max,2}$ | 1000 ms^-1^(37 °C) |  |
| Ca^2+^ diffusion coefficient | $D_{Ca}$ | 0.220 μm^2^ ms^-1^  (20-24 °C) | [25] |
| Binding rate constant of mobile buffer | $k_{bf}$ | 0.176 μM^-1^ ms^-1^ | MCMC  estimated |
| Unbinding rate constant of mobile buffer | $k_{bb}$ | 0.624 ms^-1^ |  |
| Total buffer concentration in compartment $j$ | $\left[ B \right]_{total,j}$ | 108.78 μM (spine head)  116.97 μM (dendritic shaft) |  |

#### Ca^2+^ influx

In Eq. (9), $J_{I_{Ca},j}$ is the Ca^2+^ influx by the Ca^2+^ current, mediated by the membrane ionotropic receptors (NDMAR and Voltage-dependent calcium channel (VDCC) in this study). The Ca^2+^currents at the spine head ($j=1 and 2$) and dendritic shaft ($j=4$) are assumed to be a fixed fraction of the total local input current. The fraction of the Ca^2+^ current in the total cation current through NMDARs is about 10% [19], whereas, through AMPARs, is about 0.6% [26]. Therefore, the Ca^2+^influx into the spine head and the dendritic shaft is mainly mediated by local NMDARs. We only consider the contribution of AMPARs to the depolarisation of the synaptic membrane and ignore the Ca^2+^ influx through AMPAR in this study. The Ca^2+^influx via NMDAR is given by

|  | $J_{NMDAR,i}=-\frac{f_{Ca}I_{NMDAR,i}}{Z_{Ca} F {Vol}_{j}} ,$ | (10) |
| --- | --- | --- |

where $I_{NMDAR,i}$ is the NMDAR current at site $i$,$f_{Ca}=10\%$ is the fraction of Ca^2+^ current carried by NMDAR [19], $F$= 96485.3 C Mol^−1^ is Faraday’s constant, $Z_{Ca}=2$ is the valence of Ca^2+^ ions and ${Vol}_{j}$ is the volume of compartment $j$. $J_{I_{Ca},j}$ is not valid when $j=3$ since there is no NMDAR on the spine neck.

VDCCs are located at both the spine head and the dendritic shaft. We calculate the Ca^2+^ current by VDCCs as

$$I_{VDCC,i}=G_{VDCC,i}mh\left( V_{i}-V_{r}^{{Ca}^{2+}} \right) ,$$

where $G_{VDCC,i}$ and $V_{i}$are the maximum conductance of VDCC and membrane potential at site $i$, respectively. $G_{VDCC,i}$ is estimated by MCMC. $V_{r}^{\mathrm{Ca}^{2+}}$is the membrane reversal potential of the Ca^2+^ ions

|  | $V_{r}^{{Ca}^{2+}}=\frac{RT}{Z_{ca}F}ln\frac{{Ca}_{o}}{{Ca}_{i}} ,$ | (11) |
| --- | --- | --- |

where $R$ is the ideal gas constant, $T$ is the absolute temperature in Kelvin, $Z_{ca}$is the valence of Ca^2+^ and$F$ is the Faraday constant. ${Ca}_{o}$ represents the extracellular Ca^2+^ concentration (${Ca}_{o}=$100 μM) and ${Ca}_{i}$ represents the cytosolic Ca^2+^ concentration in compartment $i$, respectively.$m$ and $h$ represent the activation and deactivation of VDCC, and they are calculated based on the following differential equations:

$$\frac{dm}{dt}=\left( 1-m \right)\alpha_{m}-m\beta_{m} ,$$

$$\frac{dh}{dt}=\left( 1-h \right)\alpha_{h}-h\beta_{h} ,$$

$$\alpha_{m}=\frac{8.5}{(1+exp(-\frac{V_{i}-8}{12.5}))} ,$$

$$\beta_{m}=\frac{35}{(1+exp(\frac{V_{i}+74}{14.5}))} ,$$

$$\alpha_{h}=\frac{0.0015}{(1+exp(\frac{V_{i}+29}{8}))} , and$$

$$\beta_{h}=\frac{0.0055}{(1+exp(-\frac{V_{m}+23}{8}))} .$$

Therefore, the Ca^2+^ influx via VDCC is given by

|  | $J_{VDCC,i}=-\frac{f_{Ca}I_{VDCC,i}}{Z_{Ca} F {Vol}_{j}} .$ |  |
| --- | --- | --- |

#### Ca^2+^ pumps and leakage

We assume that there is no ER in the dendritic spine, therefore, Ca^2+^ extrusion from the cytosol of CA1 pyramidal neurons is mainly mediated by two types of membrane pumps: the plasma membrane Ca^2+^ ATPase (PMCA) and the sodium Ca^2+^ exchanger (NCX). PMCA has about a 10-fold higher affinity for Ca^2+^ but a lower turnover rate than the NCX. We follow the model of Schiegg et al. [19] to simulate Ca^2+^ extrusion using first–order Michaelis-Menten kinetics. They are modelled as

|  | $J_{mem,j}=-\sum_{n} \frac{A_{j}}{V_{j}}{Ps}_{n}V_{max,n}\frac{\left[ {Ca}^{2+} \right]_{j}}{\left[ {Ca}^{2+} \right]_{j}+K_{d,n}}+\frac{A_{j}}{V_{j}}J_{leak,j} ,$ | (12) |
| --- | --- | --- |

where the maximum pumping velocity of pump $n, (n=1, PMCA;2, NCX)$ at compartment $j$ is calculated by the multiplication of the maximum turnover rates of Ca^2+^ of pump $n$,$V_{max,n}$, by its surface density, ${Ps}_{n}$, and the ratio of surface area to volume of compartment $j$, $\frac{A_{j}}{V_{j}}$*.* $K_{d,n}$ is the dissociation constant for pump $n$. $J_{leak,j}$ is the Ca^2+^ leakage flux via the membrane, which balances the Ca^2+^ concentration at the resting level. The densities of each type of pump at different locations are estimated using a Markov chain Monte Carlo (MCMC) from the experimental data reported by Sabatini et al. [22].

#### Diffusion

We simulate the Ca^2+^ diffusion between compartments using Fick's first law [17]. The concentration change of Ca^2+^ in compartment $j$ due to diffusion is

|  | $J_{diffusion,j}=-\frac{D_{Ca}}{V_{j}}\left[ \left( \frac{A}{\delta} \right)_{j,j-1}\left( \left[ {Ca}^{2+} \right]_{j}-\left[ {Ca}^{2+} \right]_{j-1} \right) - \left( \frac{A}{\delta} \right)_{j+1,j}\left( \left[ {Ca}^{2+} \right]_{j+1}-\left[ {Ca}^{2+} \right]_{j} \right) \right] ,$ | (13) |
| --- | --- | --- |

where $D_{Ca}$ is the diffusion coefficient of Ca^2+^, and $V_{j}$ is the volume of compartment$j$. $\left( \frac{A}{\delta} \right)_{p,q}=\frac{A_{pq}}{{\Delta l}_{pq}}$ is coupling coefficient between

compartments $p$ and $q$, where$A_{pq}$is the cross-sectional area of the two compartments and ${\Delta l}_{pq}$ is the distance between the midpoints of compartments $p$ and $q$ [27].

#### Buffering

The Ca^2+^ buffer proteins are divided into immobile and mobile ones based on their diffusion ability [28]. In each compartment, we include a general form of an endogenous immobile Ca^2+^ buffer protein and two mobile buffer proteins: calbindin (CaD) and calmodulin (CaM).The buffers capacity for Ca^2+^ decides the decay in the cytosolic Ca^2+^ concentration after reaching a peak. The fourth term in Eq. (9) is an expression for Ca^2+^ buffering by all types of endogenous buffers. The concentration of immobile buffer protein in each compartment is estimated by MCMC.

### Estimation of the number of synaptic AMPAR

Based on the experiments [29, 30], we assume that EPSP amplitude at the synaptic site is lower than 5 mV after a single synaptic stimulation. As EPSP is mainly AMPAR-dependent and partially NMDAR-dependent, we adjust the number of AMPARs to generate the expected EPSP amplitude. The range of AMPAR numbers for testing is calculated based on the spine head size and the density of AMPAR [15]. It has been reported that AMPAR density in the synaptic site in the adult cerebellum ranges from 48 to 1210 receptors per μm^2^ [31]. Therefore, we choose a large AMPAR density in PSD in this model, which causes EPSPs of 4.5 mV and 7 mV at spine and shaft, respectively (Figure E).


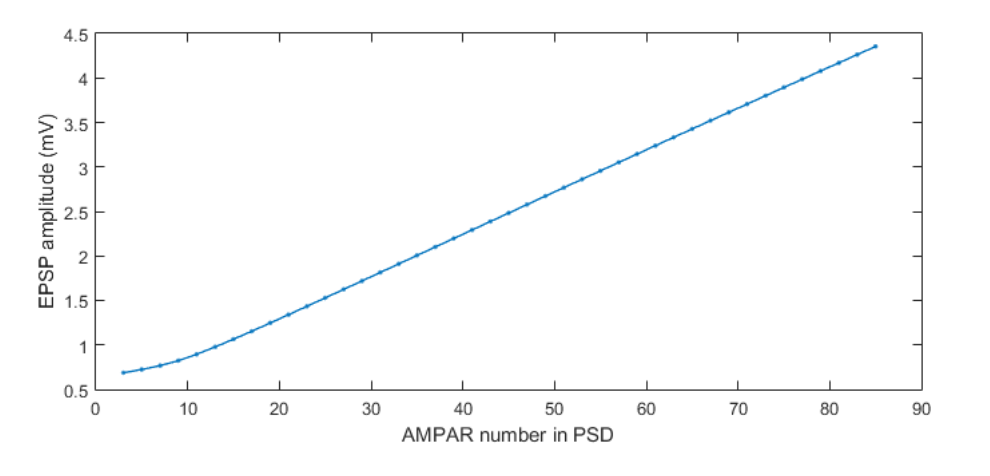


Figure E. AMPAR numbers in PSD positively correlate to EPSP amplitude in the spine head. The area of PSD in this model is 0.07 μm^2^ (r = 0.15 μm), which gives a range of AMPAR from three to 86. The neck conductance we use in the simulation is 157 MΩ, representing the resistance of a medium sized spine neck of CA1 pyramidal neurons [6].

### Parameter estimation using MCMC

We choose four target outputs from the experiments by Sabatini et al. [22]: the amplitudes of Ca^2+^ transient (${\Delta[Ca]}_{bAP}$) in response to a single backpropagation of action potentials (bAP) in the spine and dendrite, and the corresponding decay time constant $\tau_{decay}$ (Table E).

Table E. Values of target experimental data, MCMC sample of the parameter set with the lowest mean absolute percentage error (MAPE).

| **Target experimental data** | **Target value** | **Set with the lowest MAPE** |
| --- | --- | --- |
| ${\Delta[Ca]}_{bAP}$ in spine(μM) | 1.7 (±0.6) | 1.668 |
| ${\Delta[Ca]}_{bAP}$ in dendrite(μM) | 1.5 (±0.5) | 1.471 |
| $\tau_{decay}$ in spine (ms) | 12 (±4) | 11.8580 |
| $\tau_{decay}$ in dendrite (ms) | 15(±5) | 14.9430 |
| $\frac{{[Ca]}_{PMCA}}{{[Ca]}_{NCX}}$ in spine | 1.070 | 0.9213 |
| $\frac{{[Ca]}_{PMCA}}{{[Ca]}_{NCX}}$ in dendrite | 0.825 | 0.5409 |
| MAPE |  | 0.0896 |

The bAP is simulated by injecting potential into the spine head and dendrite compartment of the passive electrical model to generate amplitudes of bAPs 66.4 and 66.7 mV, respectively [32]. The simulation detail of bAP stimulation is given in next Section 7. This leads to Ca^2+^ influx through VDCC at the spine head or the dendritic shaft. According to the experimental conditions in the experiments by Sabatini et al [22], we set the glutamate concentration at the resting level (0.5 mM) to eliminate the NMDAR-dependent Ca^2+^ transient. Therefore, VDCCs provide the only Ca^2+^ source for the elevation of cytosolic Ca^2+^ level during bAP. Under this condition, the mobile buffers are significantly washed out. Therefore, we set the concentration of CaD and CaM in all compartments to zero.

The clearance of cytosolic Ca^2+^ is mediated by the endogenous protein and membrane pumps. The ability of the clearance can be evaluated by measuring the Ca^2+^ decay time constant$\tau_{decay}$. We calculate the value of$\tau_{decay}$according to the experiment of Sabatini et al. [22] by fitting a single exponential to the Ca^2+^ concentration after reaching the peak value and until t = 200 ms. The contribution of PMCA and NCX on Ca^2+^ extrusion in response to a single bAP in the dendritic spine is reported in Scheuss et al. (2006). We use the ratio of the fraction of Ca^2+^ removed by PMCA to NCX [$\frac{{[Ca]}_{PMCA}}{{[Ca]}_{NCX}}$] in the spine and dendrites from their research as an additional target data for parameter estimation (Table E).

We apply MCMC method [33, 34] to estimate values of these parameters. We use the summation of mean absolute percentage error (MAPE) to measure the goodness of fit

$$M=\frac{1}{N}\sum_{n=1}^{N} \left| \frac{x_{n}^{t}-x_{n}}{x_{n}^{t}} \right| ,$$

where N is 10, $x_{n}$and $x_{n}^{t}$ are the simulation results and target values, respectively. Based on this equation, MCMC converges to a set of parameter values with a minimum summation of MAPE. The ranges of parameters selected from the established experimental literature and the parameter set with the lowest MAPE (0.0896) are in Table F. The values of target outputs produced with the parameter set with lowest MAPE are given in Table E.

Table F. Parameter ranges for MCMC and the estimated values by MCMC.

| **Parameter** | **Range for MCMC** | **Set with the lowest MAPE** |
| --- | --- | --- |
| VDCC density in spine head | 2.23 - 4.66 μm^-2^ | 3.498 μm^-2^ |
| VDCC density in shaft | 7.29 – 14.57 μm^-2^ | 9.855 μm^-2^ |
| PMCA density in spine head | 200 - 2000 μm^-2^ | 236.554 μm^-2^ |
| PMCA density in shaft | 200 - 2000 μm^-2^ | 296.785 μm^-2^ |
| NCX density in spine head | 10 - 700 μm^-2^ | 258.991 μm^-2^ |
| NCX density in shaft | 100 -1000 μm^-2^ | 551.426 μm^-2^ |
| Immobile buffer concentration in spine head | 50 - 200 μM | 108.782 μM |
| Immobile buffer concentration in shaft | 50 - 200 μM | 116.966 μM |
| Binding rate constant of immobile buffer | 0.05 – 1 μM^-1^ms^-1^ | 0.176 μM^-1^ms^-1^ |
| Unbinding rate constant of immobile buffer | 0.05 – 1 ms^-1^ | 0.624 ms^-1^ |

### Stimulation protocol

#### Presynaptic stimulation

When the presynaptic action potential (AP) reaches the presynaptic terminal, glutamate vesicles are released into the synaptic cleft. The glutamate receptors in the membrane of the postsynaptic spine are activated by the glutamate, resulting in excitatory postsynaptic potential (EPSP). Experimentally, the presynaptic stimulation is out by placing an electrode into the testing area to deliver stimuli with certain durations and interstimulus intervals (such as methods described in [35] and [36]).

In this research, this presynaptic stimulation is simulated as the release events of glutamate vesicles, which are closely regulated by the stimulation patterns applied. There are four types of presynaptic stimulation patterns used in this research: (1) single stimulation (or 1 s of stimulation at 1 Hz; 1 pulse); (2) 1 s of low-frequency stimulation (LFS) at 10 Hz (10 pulses); (3) 1 s of high-frequency stimulation (HFS) at 100 Hz (100 pulses); and (4) theta burst stimulation (TBS) (Figure F) [37-39].

(1) single stimulation
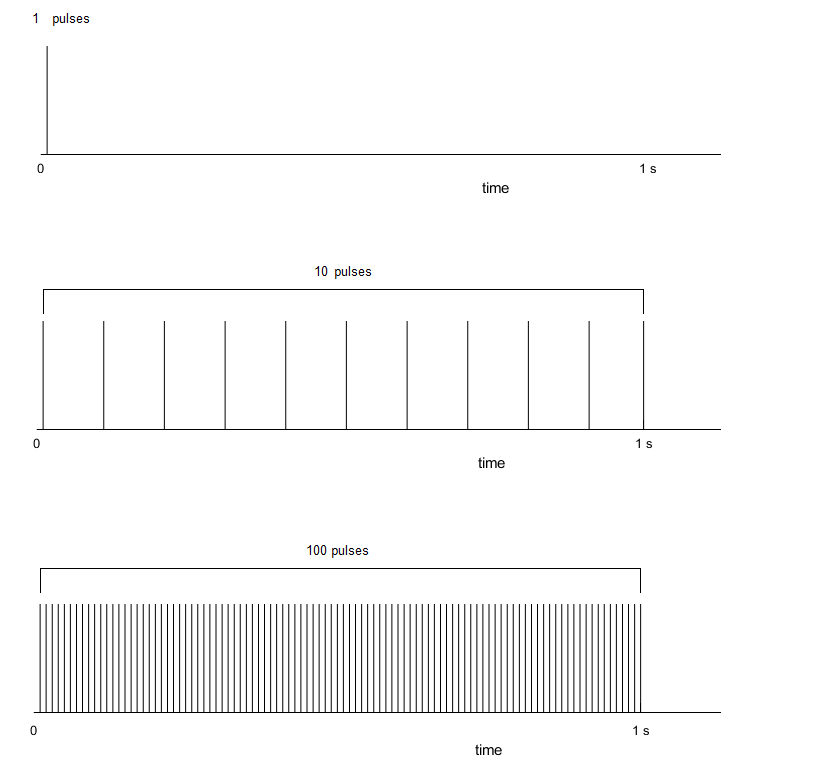


(2) LFS


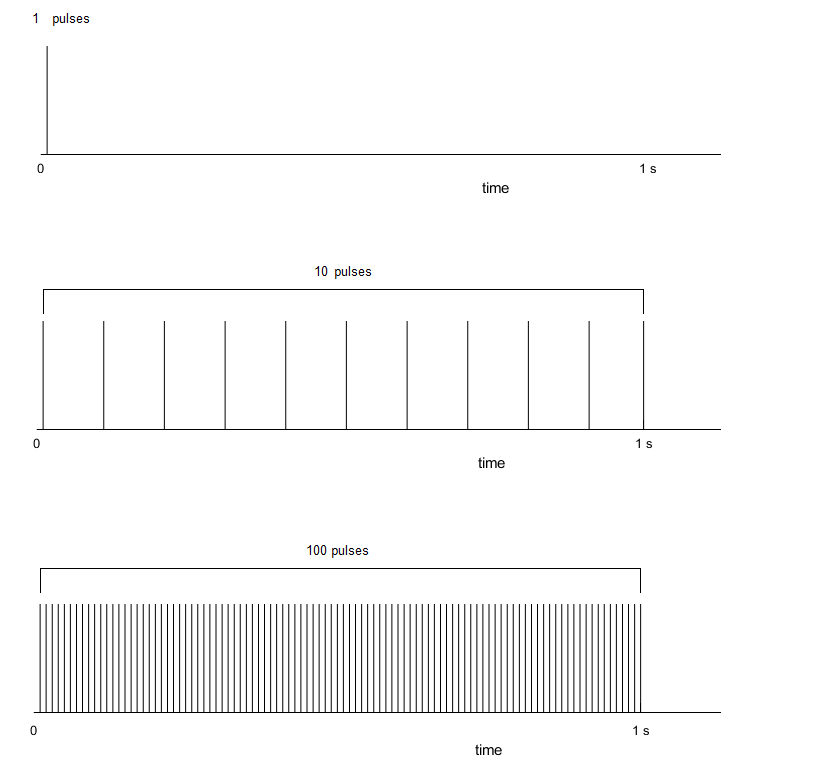


(3) HFS


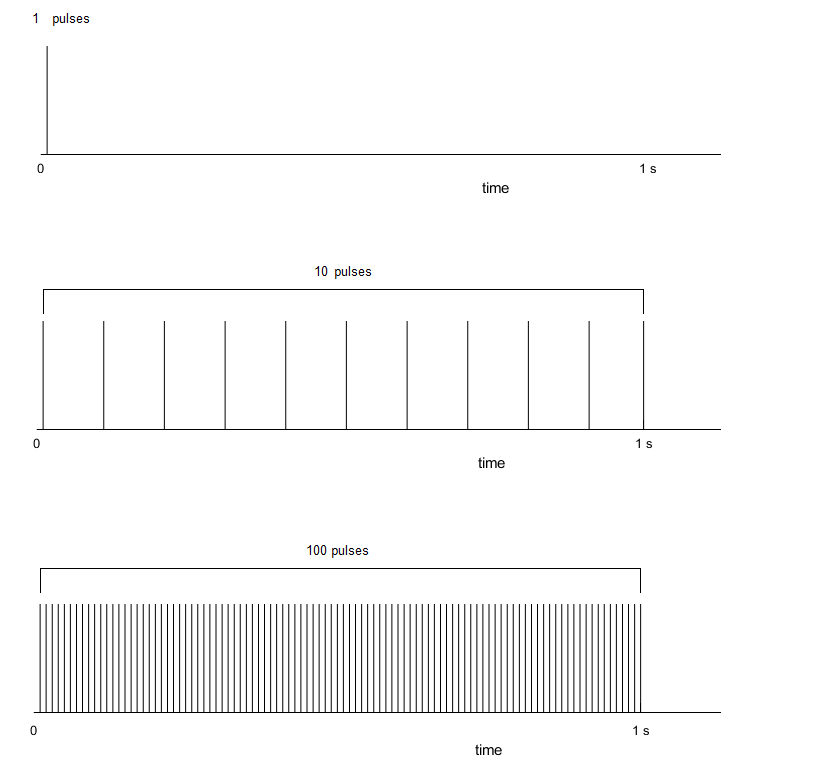


(4) TBS
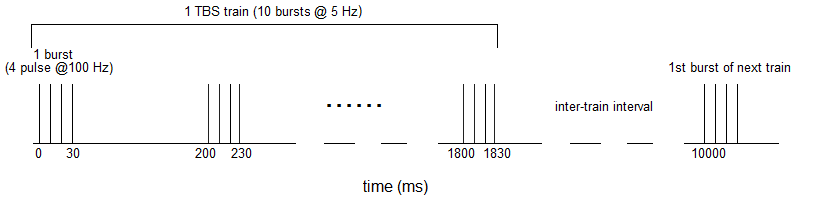


Figure F. Stimulation patterns. (1) A single pulse stimulation (or 1 s of stimulation at 1 Hz; 1 pulse); (2) 1 s of LFS at 10 Hz (10 pulses); (3) 1 s of HFS at 100 Hz (100 pulses); and (4) TBS. One TBS train consists of 10 stimulus bursts at 5 Hz and each burst consists of four pulses at 100 Hz. The TBS trains are delivered at 0.1 Hz (10 s separation between trains) [38].

#### Postsynaptic stimulation

AP of a neuron travels along the axon and it has been found that AP can propagate back to the dendrites of the pyramidal neurons. This phenomenon is called bAP. The bAP can create a strong depolarisation on the dendritic membrane, with a brief duration. It activates VDCC to induce ions flux across the membrane. We simulate the bAP by injecting a potential into the spine head and dendrite shaft to generate the bAP amplitudes of bAPs 66.4 and 66.7 mV, respectively [32].

We inject the decay of bAP is simulated by the following function:

$$V_{bAP}=A(k_{f} \theta\left( t-t_{i} \right)e^{\frac{-(t-t_{i})}{\tau_{f}}}+k_{s} \theta\left( t-t_{i} \right)e^{\frac{-(t-t_{i})}{\tau_{s}}}) ,$$

where $A$ is the bAP amplitude, $k_{f}$ = 75% and $k_{s}$ = 25% are the proportions of fast and slow decay, respectively. $\tau_{f}$= 3ms and $\tau_{s}$ = 25 ms are the time constants for fast and slow decay, respectively. $\theta\left( t-t_{i} \right)$ is a Heaviside function in which$\theta\left( st-t_{i} \right)=\left\{ \begin{aligned} 0, if t<t_{i}, \\ 1, if t\geq t_{i}. \end{aligned} \right. t$ is the current time and $t_{i}$ is the time at when bAP occurs ($t_{i}$ = 0 in this case). Parameter values are taken from [40].

#### Pairing stimulation

Both presynaptic stimulation and bAP themselves are not enough to induce significant depolarisation in the postsynaptic membrane. In the experiments, stimulation of both the presynaptic and postsynaptic neurons are used to create a pairing of the bAP and the presynaptic stimulation, which causes a large depolarisation and Ca^2+^ influx through the NMDARs [41] (Figure G). The interval between the pairing stimulation is a critical factor as it controls the significance of postsynaptic depolarisation [42].


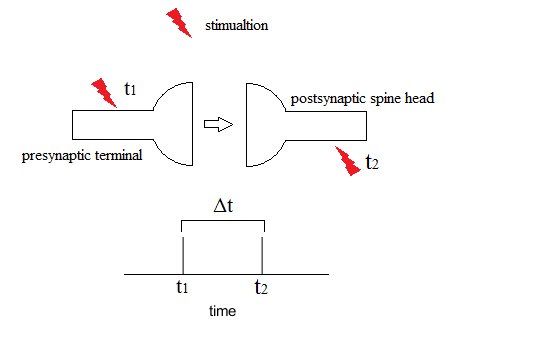


Figure G. Pairing stimulation. A pairing stimulation contains a presynaptic stimulation at time t1 and a postsynaptic stimulation at t2. The time interval between the pairing stimulation is t2 – t1 = Δt.

### Simulation: Model performance under the control condition

#### Glutamate profile and receptor activity

After release from the vesicle, the glutamates diffuse rapidly from the synaptic cleft. This gives a peak concentration of 0.7 mM and a sharp decrease to the resting level of less than 1 ms (Figure H (1) left panel). The maximum fractions of receptors opened are 0.37 (synaptic NR2A-NMDAR), 0.044 (synaptic NR2B-NMDAR) and 0.25 (synaptic AMPAR), respectively (Figure H (2) top panel). A single EPSP stimulus is too weak to activate receptors at the perisynaptic and extrasynaptic sites (Figure H (2) bottom panel) because of the large extrasynaptic volume in comparison with the synaptic cleft, and the long distance between them and the release site. Even for the presynaptic NMDARs, the local glutamate concentration peak is 33 µM (Figure H (1) middle panel), which gives a maximum open fraction lower than 0.0025 (Figure H (2) bottom left panel).

(1)


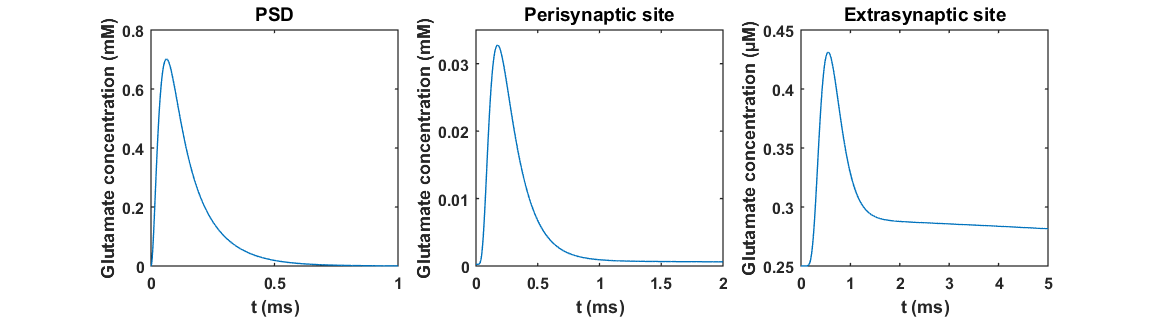


(2)


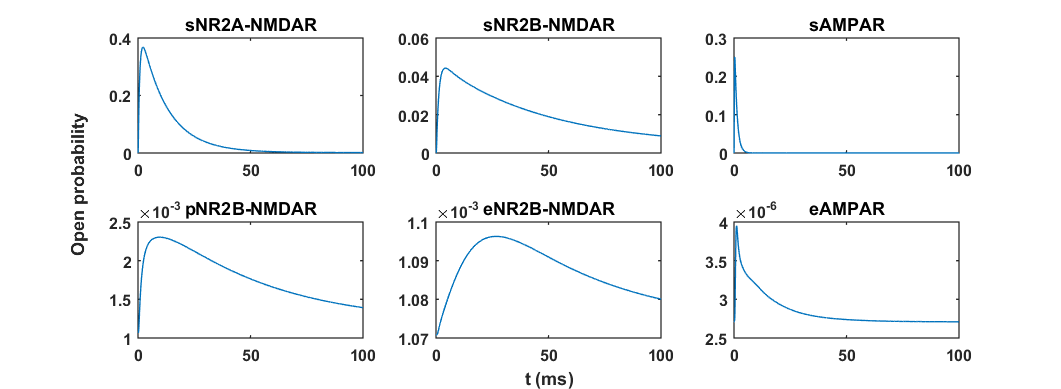


Figure H. (1) Glutamate concentration and (2) receptor response at different locations. Simulation is carried in response to a single EPSP stimulus under the control condition.

#### Postsynaptic membrane depolarisation

A single presynaptic or EPSP stimulus leads to maximums of 4.5 mV and 1.7 mV depolarisation at the spine head and the dendritic shaft, respectively (Figure I). The depolarisation levels are not strong enough to open more VDCCs than it under the resting conditions. The Ca^2+^ influx depends mostly on the opening of NMDARs by depolarisation and the glutamates diffused from the release sites.


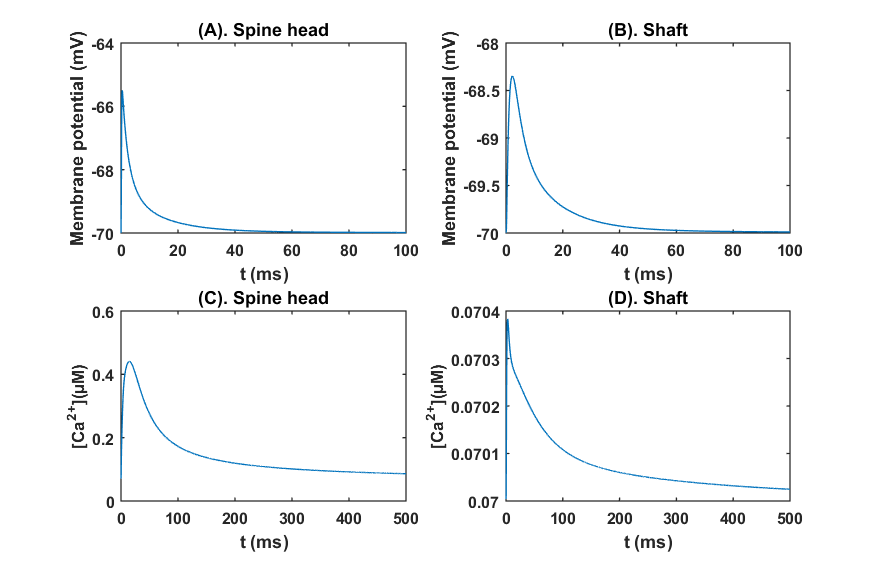


Figure I. Membrane depolarisation by a single EPSP stimulus at (1) the spine head and (2) the dendritic shaft.

#### Mobile buffer proteins

When using MCMC, we removed the mobile buffers, CaD and CaM, by setting their concentration to zero to satisfy the experimental conditions for parameter estimation. Before starting the further simulation, we need to return them back to their standard levels (Table D). In response to a single vesicle release event from the presynaptic terminal (a single EPSP stimulus), the Ca^2+^ peak amplitude in the spine head is lower (${\Delta[Ca]}_{\mathrm{EPSP}}=0.5 \mu M$) with a slower decay time in the presence of CaD and CaM than in the absence of these two buffer proteins (${\Delta[Ca]}_{\mathrm{EPSP}}=1.8 \mu M$) (Figure J). A large fraction of free Ca^2+^ ions is removed by the mobile buffers. The value of ${\Delta[Ca]}_{\mathrm{EPSP}}$ lies in the range estimated under experimental conditions [17]. A single EPSP stimulus limits the effect on cytosolic Ca^2+^ to the spine head only. The change in Ca^2+^ levels in the dendritic shaft is negligible.


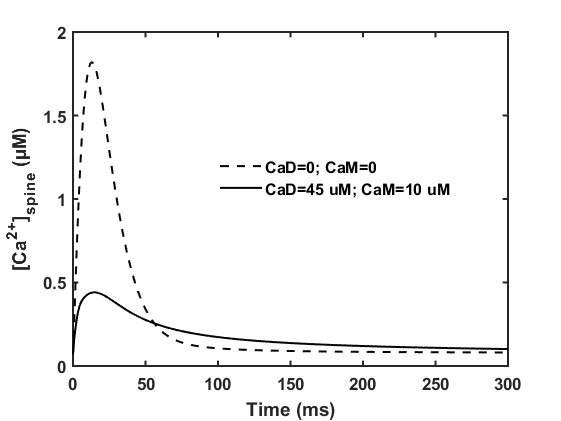


Figure J. Ca^2+^ transient in the spine head in response to a single pulse presynaptic stimulation.

#### Multipulse stimulation

We next simulate the model in response to different stimulation patterns: 1 s stimulation in LFS (10 Hz) and HFS (100 Hz) (see Section 7 for a detailed explanation). In both HFS and LFS conditions, glutamate transients at each stimulation pulse are well separated from each other because of the rapid diffusion (Figure K). The peak concentrations induced by each stimulation are at the same level, with the only exception being at the extrasynaptic site. Under HFS, the peak glutamate concentration at the extrasynaptic site increases as more stimulation pulses arrive and approaches a maximum concentration of around 0.47 µM under HFS condition (Figure K (2) right panel).

(1) 10 Hz


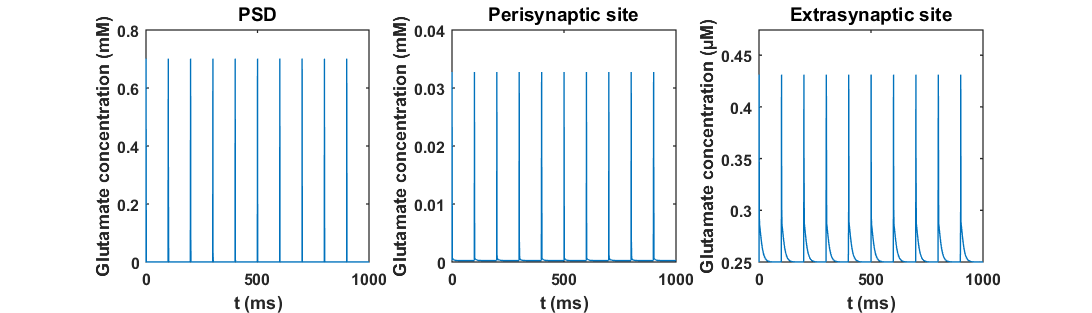


(2) 100 Hz


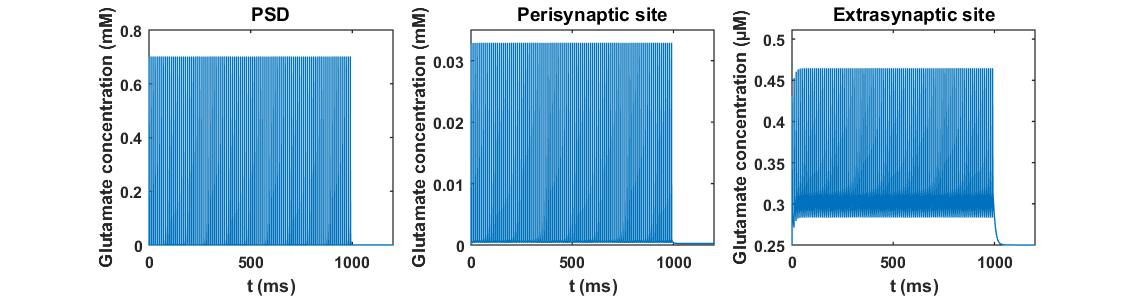


Figure K. Glutamate concentration at different locations in response to (1) 1s of LFS (10 Hz) and (2) 1s of HFS (100 Hz).

The maximum fraction of NMDARs in PSD increases in response to the first few pulses and then decreases to a lower level under both LFS and HFS (Figure L (1) and (2)). The synaptic NR2B-NMDAR shows a faster decrease in amplitude than under LFS and synaptic NR2A-NMDAR. The decrease results in the fast desensitisation of NR2B-NMDAR.

In the perisynaptic and extrasynaptic sites, a higher fraction of NMDARs open as more pulses arrives at the synapse. The maximum fraction and the increase in the peak fraction are higher in HFS than in LFS, indicating that the temporal summation of HFS allows more glutamate to accumulate after escaping from the synaptic cleft. The summation effect on glutamate concentration leads to a higher open fraction of glutamate receptors; however, it causes a significant increase in receptor desensitisation because of the prolonged exposure to higher levels of glutamate than in the resting level. Consequently, the Ca^2+^ transient in the spine head rises to a maximum of 3 µM after the first seven to eight pulses and decreases to around 1 µM before 400 ms after the start of the stimulation (Figure L (4)).

(1)

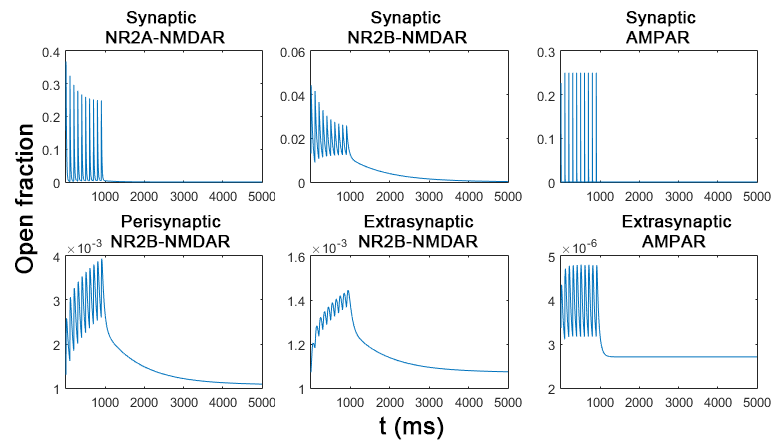


(2)

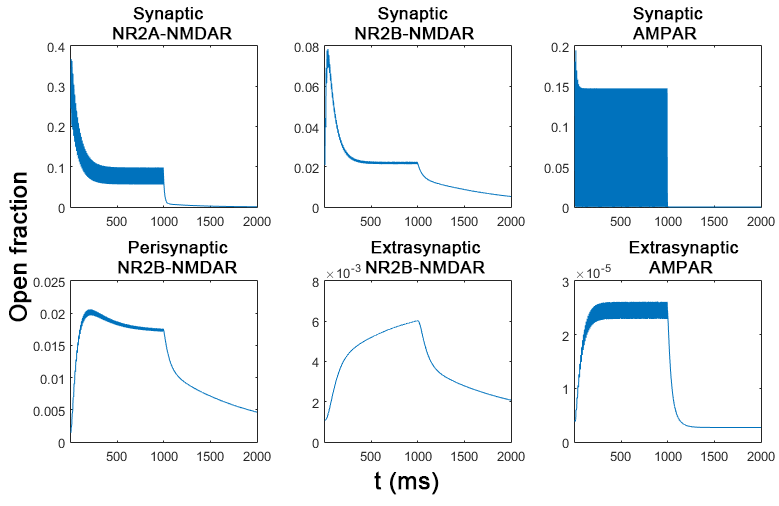

(3)

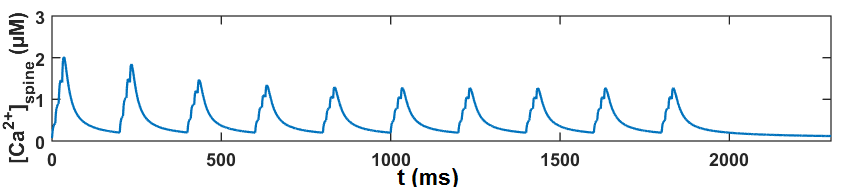

(4)

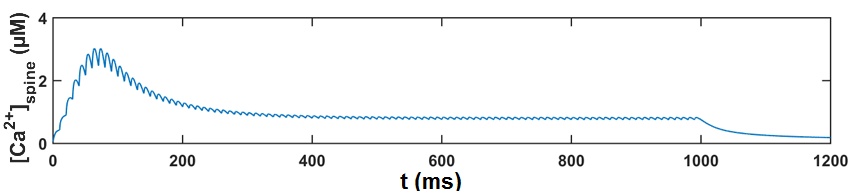


Figure L. States transition of the receptors and Ca^2+^ transient in the spine head in response to 1s of LFS (10 Hz) and 1s of HFS (100 Hz). States transition of the receptors by type and location in response to (1) LFS and (2) HFS. The Ca^2+^ ions transient in the spine head are induced by (3) LFS and (4) HFS.

### Integration of Ca^2+^ model and CaMKII ST model.

We simulate the formation of the CaMKII-NMDAR complex in PSD in response to presynaptic stimulation by modifying the CaMKII ST model developed by He, Kulasiri (43). Their model consists of a series of key events: (1) formation of Ca_4_CaM complex; (2) activation and autophosphorylation of the CaMKII subunit; (3) translocation of the CaMKII holoenzyme into PSD; and (4) the formation of CaMKII-NMDAR complex in PSD. These events are downstream events of Ca^2+^ influx via NMDAR after stimulation. We have already included the event (1) in the Ca^2+^ model (Fig 2 C). Therefore, the Ca_4_CaM complex is a link between the Ca^2+^ model and the CaMKII ST model. Besides, the other link between the two model is the postsynaptic NR2B-NMDAR, which serves as a Ca^2+^ channel in the Ca^2+^ model and as a CaMKII binding partner in the CaMKII ST model.

We apply a high-frequency presynaptic stimulation (HFS; 100 pulses at 100 Hz) and a high-frequency presynaptic stimulation paired with postsynaptic stimulation (pairing HFS; 100 pulses at 100 Hz) to our Ca^2+^ model, respectively (see Section 7 for a detailed explanation). We use a paired pre/postsynaptic stimulation protocol because experimental evidence showed that postsynaptic membrane depolarisation triggered by presynaptic stimulation alone was not sufficient to induce LTP [44, 45]. Paired stimulation at both the presynaptic and postsynaptic neurons are used to create a pairing of EPSP and bAP, which leads to a large depolarisation and Ca^2+^ elevation by NMDAR in the postsynaptic spine head [41]. When we simulate the pairing HFS protocol, a 2 ms-delayed bAP is introduced after each presynaptic stimulus pulse.

The result produced by the model of He, Kulasiri (43) did not show any desensitisation of NMDARs (Figure M (1)). This may be because of the assumptions that NMDARs are not the major Ca^2+^ channels, or that NMDARs recover from desensitisation completely between two pulses. Both of these assumptions conflict with the setting of our model that NMDARs are the major Ca^2+^ channels in the spine head and the experimental observations support that both NR2A-NMDAR and NR2B-NMDAR are desensitised under high-frequency stimulation [10]. The Ca^2+^ response of Ca^2+^ model to HFS and pairing HFS reaches a peak level, decreases, then stays on a plateau at a lower level until the end of stimulation which reflects a large desensitisation of synaptic NMDAR by high-frequency stimulation (Figure M (2)). Under HFS, the elevation in $\left[ \mathrm{Ca}^{2+} \right]_{\mathrm{cyto}}$ is much less than in the original Ca^2+^ response. In contrast, under the pairing HFS, although the peak level of $\left[ \mathrm{Ca}^{2+} \right]_{\mathrm{cyto}}$ can be above 40 μM and is higher than the maximum $\left[ \mathrm{Ca}^{2+} \right]_{\mathrm{cyto}}$ for the original Ca^2+^ response (around 20 μM), the plateau level (around 5 μM) is much lower than of the original response during the same stimulation time period.

We next compare the levels of the CaMKII-NMDAR complex in the original and our Ca^2+^ results. Before simulation, we have made several adjustments prior to connecting the Ca^2+^ model to CamKII ST model. In CaMKII ST model, all proteins are in units of particle numbers (#). The time-dependent changes in the concentration of these proteins are calculated in particle numbers and all concentration-based rate constants are in #^-1^s^-1^. Therefore, we convert the Ca_4_CaM concentration (in μΜ) from Ca^2+^ model into particle numbers (in #) before calculating the translocation of CaMKII. The conversion is according to the following formula

$$particle number =concentration \times N_{A}\times Vol ,$$

where $N_{A}$ is the Avogadro constant (6.022140857$\times$10^23^ mol^−1^) and $\mathrm{Vol}$ is the volume of the spine head (0.1fL).

The rate constants used in the CaMKII ST model are based on 37°C, therefore, we adjust them to 34°C using a Q_10_ of 2.15 [46] to be consistent with the conditions in the Ca^2+^ model. The simulation results of CaMKII ST model show a minor difference in the level of CaMKII-NMDAR complex formation at 34°C and 37°C (Figure M (3)).

Furthermore, we adjust the NR2B number in the CaMKII ST model from 20 to 8, which is the standard value based on our previous assumptions in the Ca^2+^ model. This decreases the level of the CaMKII-NMDAR complex by CaMKII ST model at t =300s by 1 (Figure M (3)). In contrast, the level of the CaMKII-NMDAR complex by our Ca^2+^ model in response to pairing HFS is about 0.7 (Figure M (4)) lower than in response to the original Ca^2+^ input (Figure M (3)). The difference is because a larger fraction of NMDARs are desensitised during pairing HFS and this leads to fewer Ca^2+^ ions entering the spine than the original input in CaMKII ST. There is no CaMKII-NMDAR formation in response to presynaptic HFS alone because of the insufficient amount of Ca^2+^ ion entered into the cytosol.

Moreover, we also use TBS as an optional stimulation protocol (See Section 7). In a train of TBS, pulses are grouped into several bursts, and the time duration (200 ms) between two bursts allows desensitised NMDARs to partially recover. TBS is considered to be a more physiologically relevant stimulus, which is close to the frequency of the endogenous hippocampal rhythm that triggers LTP [38, 47]. One train of TBS consists of 10 stimulus bursts at 5 Hz (200 ms separation between bursts) and each burst consists of four pulses at 100 Hz. A 2 ms-delayed bAP is introduced into the model after each presynaptic stimulus pulse. Four trains of TBS are delivered at 0.1 Hz (10 s separation between trains), which is used to induce LTP experimentally [38]. The changes of Ca^2+^ level in the spine head by four trains of TBS and the corresponding production of CaMKII-NMDAR complex are shown in Figure M (5) and (6), respectively. The level of the CaMKII-NMDAR complex shows a good agreement to that produced by the original Ca^2+^ input (Figure M (3)).

(1) (2)
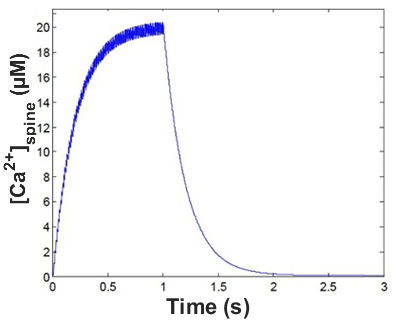

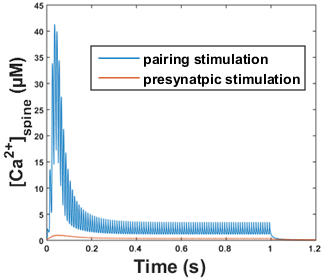

 (3) (4)

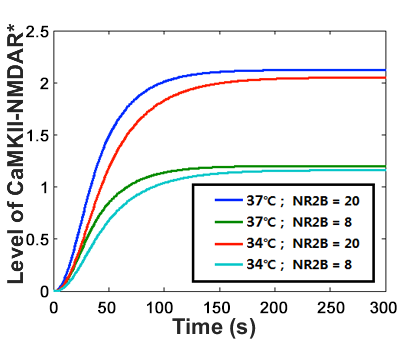

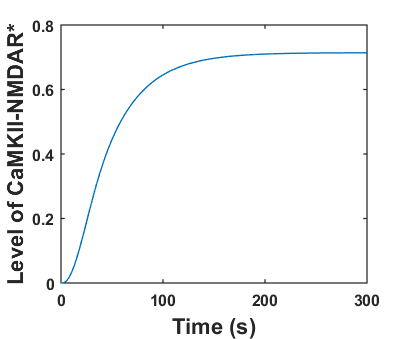

 (5) (6)

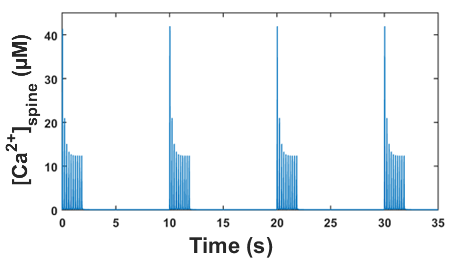

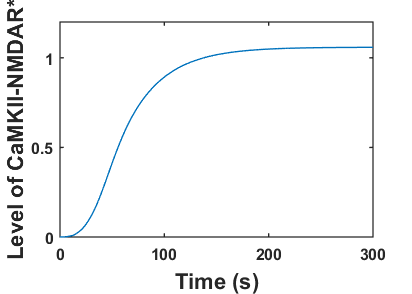


Figure M. Ca^2+^ elevation and CaMKII-NMDAR complex formation in the spine head in response to presynaptic stimulation. (1) Ca^2+^ dynamics in the spine head in response to Ca^2+^ by Eq. (9), (2) HFS and paring HFS, and (5) 4 trains of TBS, respectively, and the corresponding CaMKII-NMDAR complex production (3, 4 and 6).

### Global sensitivity analysis using PRCC

Two thousand perturbed factor sets are generated using Latin hypercube sampling (LHS) [48] according to the range in Table 4. LHS makes sure each factor is evenly distributed in the given range and effectively reduces the correlation among the factors.

We use a partial rank correlation coefficient (PRCC) to identify the most important or sensitive factors [49]. PRCC is useful to measure the nonlinear but monotonic relationships between outputs and inputs. Therefore, the factor ranges are chosen according to previous sections, to ensure that there are no non-monotonicity relationships between the selected factors and the corresponding outputs. In each realisation, we run the model with one set from the factor matrix generated by LHS while keeping other parameters at the standard values. Both the LFS and HFS conditions are simulated.

We calculate the PRCC value and the corresponding p-value for each factor against each output using a PRCC Matlab toolbox [49]. If the p-value of a PRCC result is greater than 0.05, it fails to reject the null hypothesis that no relationship exists between the factor and the output. The PRCC results are shown in Fig 9. Generally, we assume that there is a correlation between the factor and the output when the absolute PRCC value is greater than 0.5 [39]. Based on this, we modified the results and, therefore, in Fig 9, the white colour denotes that there is no correlation between corresponding factor and the output.

## Reference:

1. Rusakov DA, Kullmann DM. Extrasynaptic Glutamate Diffusion in the Hippocampus: Ultrastructural Constraints, Uptake, and Receptor Activation. The Journal of Neuroscience. 1998;18(9):3158-70.

2. Herman MA, Jahr CE. Extracellular glutamate concentration in hippocampal slice. The Journal of neuroscience : the official journal of the Society for Neuroscience. 2007;27(36):9736-41. Epub 2007/09/07. doi: 10.1523/jneurosci.3009-07.2007. PubMed PMID: 17804634; PubMed Central PMCID: PMCPmc2670936.

3. Talantova M, Sanz-Blasco S, Zhang X, Xia P, Akhtar MW, Okamoto S-i, et al. Aβ induces astrocytic glutamate release, extrasynaptic NMDA receptor activation, and synaptic loss. Proceedings of the National Academy of Sciences. 2013;110(27):E2518-E27. doi: 10.1073/pnas.1306832110.

4. Sorra KE, Harris KM. Overview on the structure, composition, function, development, and plasticity of hippocampal dendritic spines. Hippocampus. 2000;10(5):501-11.

5. Holmes WR, Levy WB. Insights into associative long-term potentiation from computational models of NMDA receptor-mediated calcium influx and intracellular calcium concentration changes. Journal of neurophysiology. 1990;63(5):1148-68. Epub 1990/05/01. PubMed PMID: 2162921.

6. Sorra KE, Harris KM. Overview on the structure, composition, function, development, and plasticity of hippocampal dendritic spines. Hippocampus. 2000;10(5):501-11. Epub 2000/11/15. doi: 10.1002/1098-1063(2000)10:5<501::aid-hipo1>3.0.co;2-t. PubMed PMID: 11075821.

7. O'Donnell C, Nolan MF, van Rossum MCW. Dendritic Spine Dynamics Regulate the Long-Term Stability of Synaptic Plasticity. The Journal of Neuroscience. 2011;31(45):16142-56. doi: 10.1523/jneurosci.2520-11.2011.

8. Rusakov DA. The role of perisynaptic glial sheaths in glutamate spillover and extracellular Ca(2+) depletion. Biophysical journal. 2001;81(4):1947-59. doi: 10.1016/S0006-3495(01)75846-8. PubMed PMID: 11566769; PubMed Central PMCID: PMC1301670.

9. Meldrum BS. Glutamate as a Neurotransmitter in the Brain: Review of Physiology and Pathology. The Journal of Nutrition. 2000;130(4):1007.

10. Erreger K, Dravid SM, Banke TG, Wyllie DJ, Traynelis SF. Subunit-specific gating controls rat NR1/NR2A and NR1/NR2B NMDA channel kinetics and synaptic signalling profiles. The Journal of physiology. 2005;563(Pt 2):345-58. Epub 2005/01/15. doi: 10.1113/jphysiol.2004.080028. PubMed PMID: 15649985; PubMed Central PMCID: PMCPmc1665591.

11. Jonas P, Major G, Sakmann B. Quantal components of unitary EPSCs at the mossy fibre synapse on CA3 pyramidal cells of rat hippocampus. The Journal of physiology. 1993;472:615-63. PubMed PMID: PMC1160505.

12. Cheng D, Hoogenraad CC, Rush J, Ramm E, Schlager MA, Duong DM, et al. Relative and Absolute Quantification of Postsynaptic Density Proteome Isolated from Rat Forebrain and Cerebellum. Molecular & Cellular Proteomics. 2006;5(6):1158-70. doi: 10.1074/mcp.D500009-MCP200.

13. Chua JJE, Kindler S, Boyken J, Jahn R. The architecture of an excitatory synapse. Journal of Cell Science. 2010;123(6):819-23. doi: 10.1242/jcs.052696.

14. Harris AZ, Pettit DL. Extrasynaptic and synaptic NMDA receptors form stable and uniform pools in rat hippocampal slices. The Journal of physiology. 2007;584(Pt 2):509-19. doi: 10.1113/jphysiol.2007.137679. PubMed PMID: PMC2277145.

15. Tanaka J-i, Matsuzaki M, Tarusawa E, Momiyama A, Molnar E, Kasai H, et al. Number and Density of AMPA Receptors in Single Synapses in Immature Cerebellum. The Journal of Neuroscience. 2005;25(4):799-807. doi: 10.1523/jneurosci.4256-04.2005.

16. Koch C. Biophysics of computation: information processing in single neurons: Oxford university press; 1998.

17. Sterratt D, Graham B, Gillies A, Willshaw D. Principles of computational modelling in neuroscience: Cambridge University Press; 2011.

18. Mainen ZF, Carnevale NT, Zador AM, Claiborne BJ, Brown TH. Electrotonic architecture of hippocampal CA1 pyramidal neurons based on three-dimensional reconstructions. Journal of neurophysiology. 1996;76(3):1904-23. Epub 1996/09/01. PubMed PMID: 8890303.

19. Schiegg A, Gerstner W, Ritz R, van Hemmen JL. Intracellular Ca2+ stores can account for the time course of LTP induction: a model of Ca2+ dynamics in dendritic spines. Journal of neurophysiology. 1995;74(3):1046-55. Epub 1995/09/01. PubMed PMID: 7500131.

20. Spruston N, Jonas P, Sakmann B. Dendritic glutamate receptor channels in rat hippocampal CA3 and CA1 pyramidal neurons. The Journal of physiology. 1995;482(Pt 2):325-52. PubMed PMID: PMC1157732.

21. Jahr CE, Stevens CF. Voltage dependence of NMDA-activated macroscopic conductances predicted by single-channel kinetics. The Journal of neuroscience : the official journal of the Society for Neuroscience. 1990;10(9):3178-82. Epub 1990/09/01. PubMed PMID: 1697902.

22. Sabatini BL, Oertner TG, Svoboda K. The Life Cycle of Ca2+ Ions in Dendritic Spines. Neuron. 2002;33(3):439-52. doi: <http://dx.doi.org/10.1016/S0896-6273(02)00573-1>.

23. Blaustein MP, Lederer WJ. Sodium/calcium exchange: its physiological implications. Physiological reviews. 1999;79(3):763-854. Epub 1999/07/03. PubMed PMID: 10390518.

24. Carafoli E. The Ca2+ pump of the plasma membrane. The Journal of biological chemistry. 1992;267(4):2115-8. Epub 1992/02/05. PubMed PMID: 1310307.

25. Allbritton NL, Meyer T, Stryer L. Range of messenger action of calcium ion and inositol 1,4,5-trisphosphate. Science (New York, NY). 1992;258(5089):1812-5. Epub 1992/12/11. PubMed PMID: 1465619.

26. Garaschuk O, Schneggenburger R, Schirra C, Tempia F, Konnerth A. Fractional Ca2+ currents through somatic and dendritic glutamate receptor channels of rat hippocampal CA1 pyramidal neurones. The Journal of physiology. 1996;491(Pt 3):757-72. PubMed PMID: PMC1158816.

27. Reeke GN, Poznanski RR, Lindsay KA, Rosenberg JR, Sporns O. Modeling in the neurosciences: from biological systems to neuromimetic robotics: CRC Press; 2005.

28. Matthews EA, Dietrich D. Buffer mobility and the regulation of neuronal calcium domains. Frontiers in cellular neuroscience. 2015;9:48.

29. Kovalchuk Y, Eilers J, Lisman J, Konnerth A. NMDA Receptor-Mediated Subthreshold Ca^2+^ Signals in Spines of Hippocampal Neurons. The Journal of Neuroscience. 2000;20(5):1791-9.

30. Palmer LM, Stuart GJ. Membrane Potential Changes in Dendritic Spines during Action Potentials and Synaptic Input. The Journal of Neuroscience. 2009;29(21):6897-903. doi: 10.1523/jneurosci.5847-08.2009.

31. Masugi-Tokita M, Tarusawa E, Watanabe M, Molnar E, Fujimoto K, Shigemoto R. Number and density of AMPA receptors in individual synapses in the rat cerebellum as revealed by SDS-digested freeze-fracture replica labeling. J Neurosci. 2007;27(8):2135-44. Epub 2007/02/23. doi: 10.1523/jneurosci.2861-06.2007. PubMed PMID: 17314308.

32. Palmer LM, Stuart GJ. Membrane potential changes in dendritic spines during action potentials and synaptic input. The Journal of neuroscience : the official journal of the Society for Neuroscience. 2009;29(21):6897-903. Epub 2009/05/29. doi: 10.1523/jneurosci.5847-08.2009. PubMed PMID: 19474316.

33. Haario H, Saksman E, Tamminen J. An adaptive Metropolis algorithm. 2001:223-42.

34. Haario H, Laine M, Mira A, Saksman E. DRAM: Efficient adaptive MCMC. Statistics and Computing. 16(4):339-54. doi: 10.1007/s11222-006-9438-0.

35. Li S, Jin M, Koeglsperger T, Shepardson NE, Shankar GM, Selkoe DJ. Soluble Abeta oligomers inhibit long-term potentiation through a mechanism involving excessive activation of extrasynaptic NR2B-containing NMDA receptors. J Neurosci. 2011;31(18):6627-38. Epub 2011/05/06. doi: 10.1523/jneurosci.0203-11.2011. PubMed PMID: 21543591; PubMed Central PMCID: PMCPmc3100898.

36. Sanhueza M, Fernandez-Villalobos G, Stein IS, Kasumova G, Zhang P, Bayer KU, et al. Role of the CaMKII/NMDA receptor complex in the maintenance of synaptic strength. Journal of Neuroscience. 2011;31(25):9170-8.

37. Kumar A. Long-term potentiation at CA3–CA1 hippocampal synapses with special emphasis on aging, disease, and stress. Frontiers in aging neuroscience. 2011;3:7.

38. Lee H-K, Barbarosie M, Kameyama K, Bear MF, Huganir RL. Regulation of distinct AMPA receptor phosphorylation sites during bidirectional synaptic plasticity. Nature. 2000;405(6789):955-9.

39. Kulasiri D, Liang J, He Y, Samarasinghe S. Global sensitivity analysis of a model related to memory formation in synapses: Model reduction based on epistemic parameter uncertainties and related issues. Journal of Theoretical Biology. 2017;419:116-36.

40. De Schutter E, Bower JM. An active membrane model of the cerebellar Purkinje cell II. Simulation of synaptic responses. Journal of neurophysiology. 1994;71(1):401-19.

41. Caporale N, Dan Y. Spike timing-dependent plasticity: a Hebbian learning rule. Annual review of neuroscience. 2008;31:25-46. Epub 2008/02/16. doi: 10.1146/annurev.neuro.31.060407.125639. PubMed PMID: 18275283.

42. Shouval HZ, Bear MF, Cooper LN. A unified model of NMDA receptor-dependent bidirectional synaptic plasticity. Proceedings of the National Academy of Sciences. 2002;99(16):10831-6. doi: 10.1073/pnas.152343099.

43. He Y, Kulasiri D, Samarasinghe S. Modelling the dynamics of CaMKII–NMDAR complex related to memory formation in synapses: The possible roles of threonine 286 autophosphorylation of CaMKII in long term potentiation. Journal of theoretical biology. 2015;365:403-19.

44. Pike FG, Meredith RM, Olding AWA, Paulsen O. Postsynaptic bursting is essential for ‘Hebbian’ induction of associative long-term potentiation at excitatory synapses in rat hippocampus. The Journal of physiology. 1999;518(Pt 2):571-6. doi: 10.1111/j.1469-7793.1999.0571p.x. PubMed PMID: PMC2269446.

45. Mansvelder HD, McGehee DS. Long-Term Potentiation of Excitatory Inputs to Brain Reward Areas by Nicotine. Neuron. 2000;27(2):349-57. doi: <http://dx.doi.org/10.1016/S0896-6273(00)00042-8>.

46. Chiba H, Schneider NS, Matsuoka S, Noma A. A simulation study on the activation of cardiac CaMKII δ-isoform and its regulation by phosphatases. Biophysical journal. 2008;95(5):2139-49.

47. Raymond CR. LTP forms 1, 2 and 3: different mechanisms for the ‘long’ in long-term potentiation. Trends in Neurosciences. 2007;30(4):167-75. doi: <http://dx.doi.org/10.1016/j.tins.2007.01.007>.

48. McKay MD, Beckman RJ, Conover WJ. A comparison of three methods for selecting values of input variables in the analysis of output from a computer code. Technometrics. 2000;42(1):55-61.

49. Marino S, Hogue IB, Ray CJ, Kirschner DE. A Methodology For Performing Global Uncertainty And Sensitivity Analysis In Systems Biology. Journal of theoretical biology. 2008;254(1):178-96. doi: 10.1016/j.jtbi.2008.04.011. PubMed PMID: PMC2570191.
